# FarmGTEx TWAS-server: An Interactive Web Server for Customized TWAS Analysis

**DOI:** 10.1093/gpbjnl/qzaf006

**Published:** 2025-02-11

**Authors:** Zhenyang Zhang, Zitao Chen, Jinyan Teng, Shuli Liu, Qing Lin, Jun Wu, Yahui Gao, Zhonghao Bai, FarmGTEx Consortium, Lingzhao Fang, Zhonghao Bai, Zhe Zhang, Jinyan Teng, Qing Lin, Jun Wu, Zhenyang Zhang, Zitao Chen, Qishan Wang, Yuchun Pan, Shuli Liu, George Liu, Yahui Gao, Bingjie Li, Bingjie Li, George Liu, Zhe Zhang, Yuchun Pan, Zhe Zhang, Lingzhao Fang, Qishan Wang

**Affiliations:** Department of Animal Science, College of Animal Sciences, Zhejiang University, Hangzhou 310058, China; Department of Animal Science, College of Animal Sciences, Zhejiang University, Hangzhou 310058, China; Guangdong Laboratory of Lingnan Modern Agriculture, Guangdong Provincial Key Lab of Agro-Animal Genomics and Molecular Breeding, College of Animal Science, South China Agricultural University, Guangzhou 510642, China; School of Life Sciences, Westlake University, Hangzhou 310024, China; Westlake Laboratory of Life Sciences and Biomedicine, Hangzhou 310030, China; Guangdong Laboratory of Lingnan Modern Agriculture, Guangdong Provincial Key Lab of Agro-Animal Genomics and Molecular Breeding, College of Animal Science, South China Agricultural University, Guangzhou 510642, China; Guangdong Laboratory of Lingnan Modern Agriculture, Guangdong Provincial Key Lab of Agro-Animal Genomics and Molecular Breeding, College of Animal Science, South China Agricultural University, Guangzhou 510642, China; Animal Genomics and Improvement Laboratory, Henry A. Wallace Beltsville Agricultural Research Center, Agricultural Research Service, USDA, Beltsville, MD 20705, USA; Department of Animal and Avian Sciences, University of Maryland, College Park, Baltimore, MD 20742, USA; Center for Quantitative Genetics and Genomics, Aarhus University, Aarhus 8000, Denmark; Scotland's Rural College (SRUC), Roslin Institute Building, Midlothian, EH25 9RG, UK; Animal Genomics and Improvement Laboratory, Henry A. Wallace Beltsville Agricultural Research Center, Agricultural Research Service, USDA, Beltsville, MD 20705, USA; Department of Animal Science, College of Animal Sciences, Zhejiang University, Hangzhou 310058, China; Department of Animal Science, College of Animal Sciences, Zhejiang University, Hangzhou 310058, China; Hainan Institute, Zhejiang University, Yongyou Industrial Park, Yazhou Bay Sci-Tech City, Sanya 572000, China; Guangdong Laboratory of Lingnan Modern Agriculture, Guangdong Provincial Key Lab of Agro-Animal Genomics and Molecular Breeding, College of Animal Science, South China Agricultural University, Guangzhou 510642, China; Center for Quantitative Genetics and Genomics, Aarhus University, Aarhus 8000, Denmark; Department of Animal Science, College of Animal Sciences, Zhejiang University, Hangzhou 310058, China; Hainan Institute, Zhejiang University, Yongyou Industrial Park, Yazhou Bay Sci-Tech City, Sanya 572000, China; Key Laboratory of Livestock and Poultry Resources Evaluation and Utilization, Ministry of Agriculture and Rural Affairs, Hangzhou 310030, China; Key Laboratory of Dairy Cow Genetic Improvement and Milk Quality Research of Zhejiang Province, Hangzhou 310030, China

**Keywords:** Transcriptome-wide association study, Farm animal, Cross species, Web server, Gene expression

## Abstract

Transcriptome-wide association study (TWAS) is a powerful approach for investigating the molecular mechanisms linking genetic loci to complex phenotypes. However, the complexity of the TWAS analytical pipeline, including the construction of gene expression reference panels, gene expression prediction, and association analysis using data from genome-wide association studies (GWASs), poses challenges for genetic studies in many species. In this study, we provide the Farm Animal Genotype-Tissue Expression (FarmGTEx) TWAS-server, an interactive and user-friendly multispecies platform designed to streamline the translation of genetic findings across tissues and species. The server incorporates gene expression data from 49 human tissues (838 individuals), 34 pig tissues (5457 individuals), and 23 cattle tissues (4889 individuals), providing prediction models for 38,180 human genes, 21,037 pig genes, and 17,942 cattle genes. It supports genotype-based gene expression prediction, GWAS summary statistics imputation, customizable TWAS analysis, functional annotation, and result visualization. Additionally, we provide 479,203, 1208, and 657 tissue–gene–trait associations for 1129 human traits, 41 cattle traits, and 11 pig traits, respectively. Utilizing the TWAS-server, we validated the association of the *ABCD4* gene with pig teat number. Furthermore, we identified that pig backfat thickness may share genetic similarities with human diastolic blood pressure, sarcoidosis (Löfgren syndrome), and body mass index. The FarmGTEx TWAS-server offers a comprehensive and accessible platform for researchers to perform TWAS analyses across tissues and species. It is freely available at https://twas.farmgtex.org, with regular updates planned as the FarmGTEx project expands to include more species.

## Graphical abstract

**Figure:**
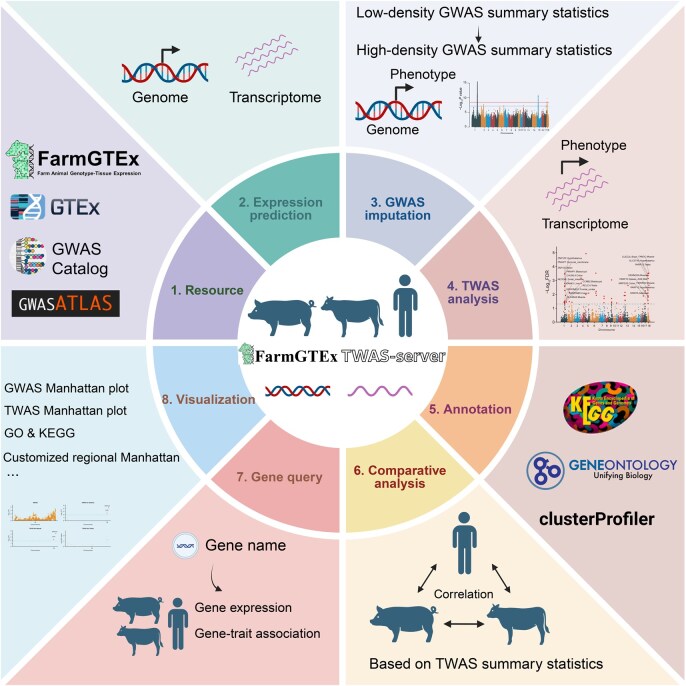

## Teaser

**Highlights**
 

• FarmGTEx TWAS-server provides an online interactive platform for transcriptome-wide association study (TWAS) analyses in humans, pigs, and cattle.
• It incorporates multiple TWAS models for 49, 34, and 23 tissues in humans, pigs and cattle, respectively.
• Comparative TWAS analyses reveal genetic similarities between porcine backfat thickness and human diastolic blood pressure, sarcoidosis, and body mass index (BMI).

## Introduction

Numerous genetic variations associated with complex diseases and traits have been discovered in human and livestock populations by genome-wide association studies (GWASs) [1–4]. However, most of these variants are located in non-coding regions and exhibit high linkage disequilibrium with other variants, making it difficult to elucidate their underlying molecular mechanisms. The integration of multi-omics data has advanced our understanding of the mechanisms by which non-coding variants contribute to complex phenotypes. Among these approaches, transcriptome-wide association studies (TWASs) have gained widespread use [5]. TWAS involves deriving gene expression prediction models from a reference panel of genotypes and gene expression data using regression or nonparametric methods. These models are then used to predict gene expression levels in individuals from GWAS cohorts based on their genotypes. By associating the predicted expression levels — representing the genetically regulated component of gene expression — with phenotypes of interest, TWAS enables the identification of gene–phenotype associations [5]. Various TWAS software packages, such as PrediXcan/S-PrediXcan [5], FUSION [6], unified test for molecular signatures (UTMOST) [7], Mendelian randomization-joint-tissue imputation (MR-JTI) [8], Transcriptome-Integrated Genetic Association Resource (TIGAR) [9], and Prediction Using Models Informed by Chromatin conformations and Epigenomics (PUMICE+) [10], have been developed to facilitate these analyses.

In human genetics, resources such as Genotype-Tissue Expression (GTEx) [11] have provided valuable gene expression reference panels across a range of tissues in hundreds of individuals. These resources have enabled the systematic investigation of regulatory effects on complex traits and diseases through TWAS [12–17]. Furthermore, various web servers, including webTWAS [18], TWAS hub [19], and TWAS Atlas [20], are available for TWAS analyses and output sharing in humans. In contrast, TWAS studies in livestock species remain underdeveloped. The Farm Animal Genotype-Tissue Expression (FarmGTEx, https://www.farmgtex.org/) project has established a transcriptome reference panel across a wide range of tissues in farm animal species, including cattle [21] and pigs [22], but the application of TWAS in these species remains limited. This gap is partly due to the complexity of TWAS analysis, which can be time-consuming and challenging for researchers without expertise in bioinformatics and statistical genetics. Additionally, the FarmGTEx datasets currently offer expression quantitative trait loci (eQTLs) exclusively trained by the Elastic Net model. While this model performs well in human studies, alternative models may be more suitable for livestock species. However, training eQTL models using multiple methods is computationally intensive.

Moreover, cross-species genetic research is crucial for understanding the evolutionary, biological, and genetic mechanisms underlying complex traits. Studies have shown that orthologous genetic variants can have conserved effects on gene expression and complex traits across species, including humans and livestock [23–29]. These findings support the potential for using polygenic transcriptomic risk scores to translate genetic signals across human populations and even across species. Furthermore, it has been suggested that livestock species would make good models for research in human biology and medicine. For instance, pigs share similarities with humans in body size, organ size, physiology, and anatomy [30], making it a suitable biological model for drug development and organ xenotransplantation in human medical research [31,32]. Therefore, combining studies in human genetics and farm animals will be important for understanding the molecular and evolutionary basis of complex phenotypes across species.

Herein, we developed the FarmGTEx TWAS-server, a user-friendly web server designed to enable TWAS analyses across multiple species, including humans, pigs, and cattle. The server facilitates cross-species translation of genetic findings by integrating three widely used TWAS software packages: S-PrediXcan [5], FUSION [6], and UTMOST [7]. Users can easily upload GWAS summary statistics to perform TWAS analyses. Additional features include LiftOver, GWAS summary statistics imputation, gene set enrichment analysis (GSEA), and result visualization. The server also provides TWAS summary statistics for various complex traits in humans, cattle, and pigs. The FarmGTEx TWAS-server is freely accessible at https://twas.farmgtex.org and will be regularly updated to include additional species as the FarmGTEx project expands.

## Results

### Overview of the FarmGTEx TWAS-server

**Figure 1:**
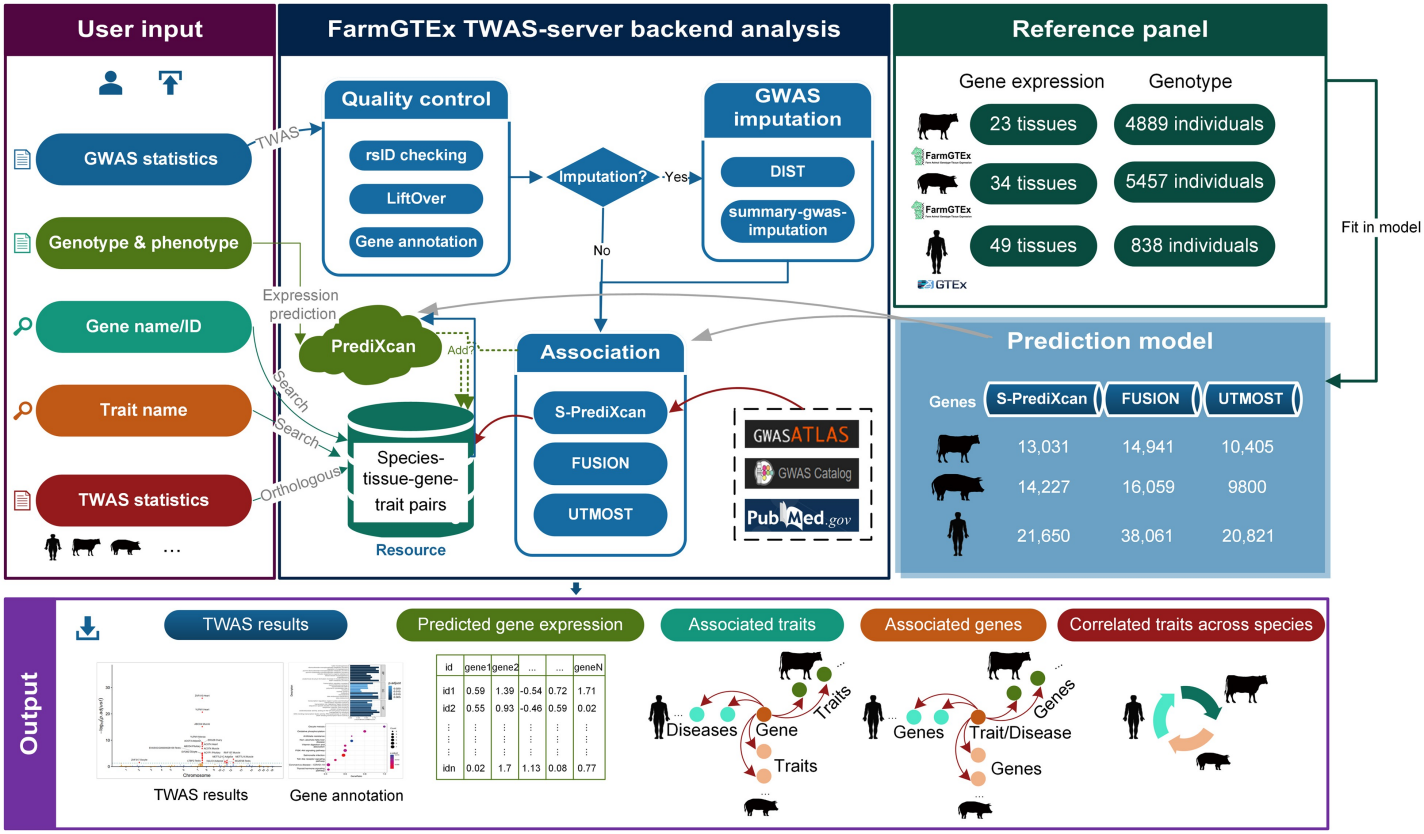
Overview of the FarmGTEx TWAS-server The FarmGTEx TWAS-server (https://twas.farmgtex.org) workflow is shown. The “Reference panel”section represents the gene expression and genotype data used for building the gene expression prediction model. The “Prediction model” section shows the number of expressed genes used in the corresponding software for each species. The colored boxes in the “User input” section correspond to their respective “Output” results, maintaining consistency in the visual representation. The label on the line connecting the “User input” and “FarmGTEx TWAS-server backend analysis” indicates the corresponding module name. The arrows point to the software or dataset employed. The TWAS-server accepts various input types: GWAS summary statistics (in blue), individual genotype/phenotype data (in green), gene names/IDs (in cyan), trait names (in orange), and TWAS summary statistics (in red). The corresponding outputs include TWAS results (in blue), predicted gene expression levels (in green), traits associated with genes (in cyan), genes associated with traits (in orange), and correlated traits across species based on the TWAS summary statistics (in red). GWAS, genome-wide association study; TWAS, transcriptome-wide association study; FarmGTEx, Farm Animal Genotype-Tissue Expression; DIST, direct imputation of summary statistics.

**Figure 1** presents an overview of the FarmGTEx TWAS-server, while **Table 1** outlines the datasets utilized. In summary, the dataset encompasses 34 pig tissues, 23 cattle tissues, and 49 human tissues. The human donors predominantly are of European ancestry, while the pig and cattle samples include individuals from various breeds worldwide. Gene expression prediction models were trained in single-tissue and multi-tissue manners based on the gene expression reference panels from the FarmGTEx [21,22] and human GTEx [11] projects. The TWAS-server can accept GWAS summary statistics and individual genotypes as input, generating predicted gene expression and TWAS results as output. Users can also compare their TWAS summary statistics with those already incorporated into the server.

**Table 1:** The datasets used in the TWAS-server

Species	Type	Description	Module	Source
Pig	Genotype	1602 WGS genotypes	GWAS imputation (panel)	FarmGTEx
	Gene expression	34 tissues (26,908 genes)	Search	
	*Cis*-eQTL models	Elastic Net, Top1, BLUP, BSLMM, CTIMP	TWAS analysis	TWAS-server, FarmGTEx
	GWAS/TWAS summary statistics	11 traits (657 significant disease–tissue–gene trios)	Orthologous, Search	TWAS-server
Cattle	Genotype	7394 WGS genotypes (SNP calling from RNA-seq); 983 WGS genotypes; 2976 WGS genotypes	GWAS imputation (panel)	FarmGTEx, AGIDB, BGVD
	Gene expression	23 tissues (27,537 genes)	Search	
	*Cis*-eQTL models	Elastic Net, Top1, BLUP, BSLMM, CTIMP	TWAS analysis	TWAS-server, FarmGTEx
	GWAS/TWAS summary statistics	41 traits (1208 significant disease–tissue–gene trios)	Orthologous, Search	Previous publication, TWAS-server
Human	Genotype	500 Europeans	GWAS imputation (panel)	https://zenodo.org/records/3657902#.Xj2Zh-F7m90
	Gene expression	49 tissues	Search	GTEx
	*Cis*-eQTL models	Elastic Net, Top1, LASSO, CTIMP	TWAS analysis	https://zenodo.org/record/3519321/ for Elastic Net http://gusevlab.org/projects/fusion/ for FUSION https://zenodo.org/record/3842289 for UTMOST
	GWAS/TWAS summary statistics	1129 traits (479,203 significant disease–tissue–gene trios)	Orthologous, Search	GWAS Catalog, webTWAS, Neale Lab UK Biobank

*Note*: Description provides attributes of the datasets, including data type, tissue, and species. Module indicates the specific module that utilizes the corresponding data (*e.g.*, GWAS imputation and TWAS analysis). Source refers to the origin of the data, such as genome reference panels or databases like GTEx or FarmGTEx. The TWAS-server specifies the data generated in this study. In particular, the models required for FUSION (Top1, BLUP, and BSLMM) and UTMOST (CTIMP) for pigs and cattle were trained as part of this research. WGS, whole-genome sequencing; GWAS, genome-wide association study; TWAS, transcriptome-wide association study; FarmGTEx, Farm Animal Genotype-Tissue Expression; BLUP, best linear unbiased prediction; BSLMM, Bayesian sparse linear mixed model; CTIMP, cross-tissue gene expression imputation; eQTL, expression quantitative trait locus; RNA-seq, RNA sequencing; LASSO, least absolute shrinkage and selection operator; SNP, single nucleotide polymorphism; GTEx, Genotype-Tissue Expression; AGIDB, animal genotype imputation database; BGVD, bovine genome variation database.

### Prediction models of gene expression

**Figure 2:**
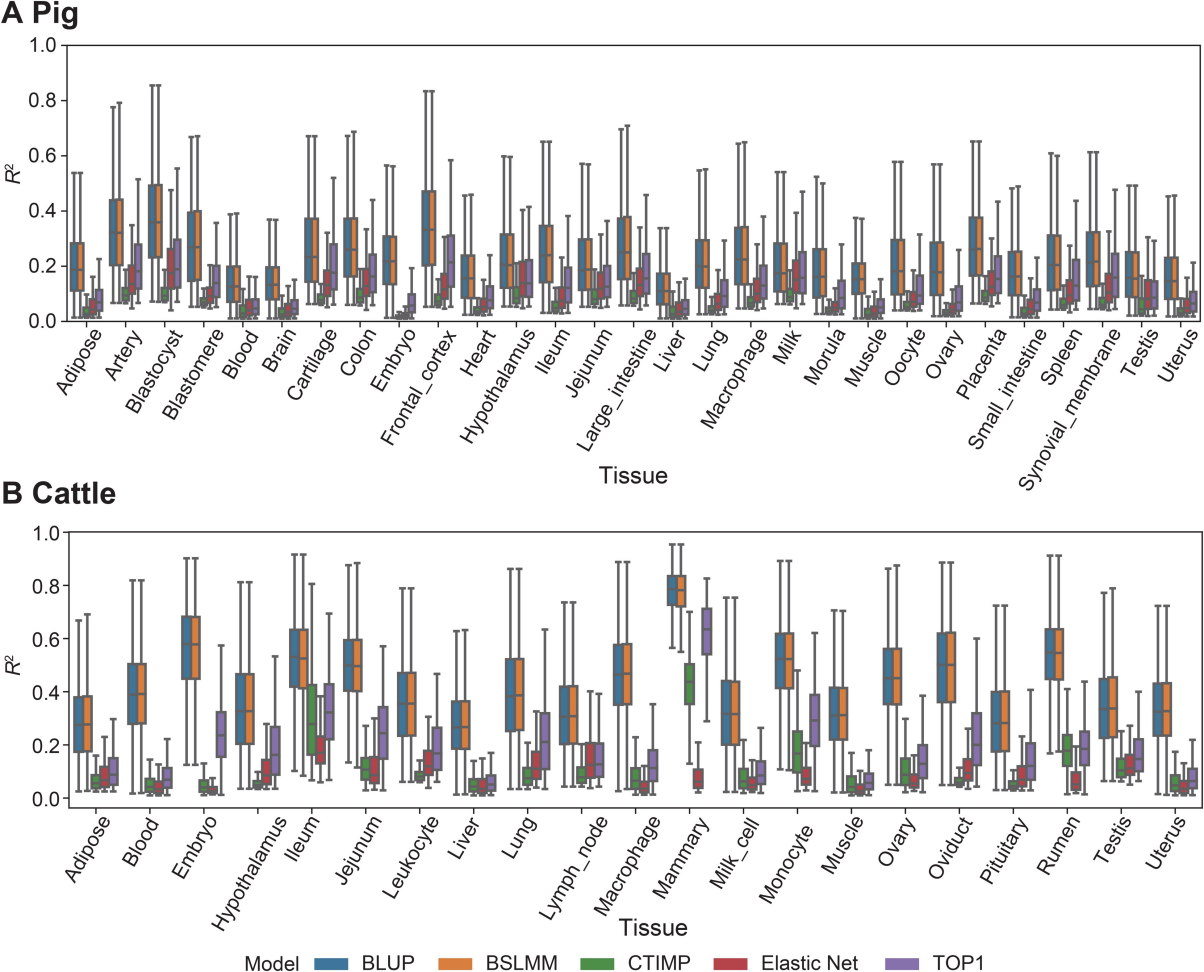
Prediction performance comparison of differrent prediction models The prediction performance of differenct models was evaluated using the squared Pearson correlation coefficient (*R*^2^) through a 5-fold cross-validation approach. **A**. Box plot showing the prediction performance of differenct models across various pig tissues. **B**. Box plot showing the prediction performance of differenct models across various cattle tissues. BLUP, best linear unbiased prediction; BSLMM, Bayesian sparse linear mixed model; CTIMP, cross-tissue gene expression imputation.

The FarmGTEx TWAS-server provides gene expression prediction models for 34, 23, and 49 tissues from pigs, cattle, and humans, respectively. Specifically, we offer the S-PrediXcan model (using Elastic Net model), FUSION models [TOP1, best linear unbiased prediction (BLUP), and Bayesian sparse linear mixed model (BSLMM)], and the UTMOST model [using cross-tissue gene expression imputation (CTIMP) model]. The models required for FUSION and UTMOST for pigs and cattle were trained as part of this study. These models allow researchers to predict gene expression levels based on genetic data for the respective species and tissues. The sample size, the number of genes with significant eQTLs (eGenes), and the significant variants (eVariants, variants associated with at least one gene’s expression) for each tissue are summarized in Tables S1–S3. The average sample size of each tissue is 184.91, 289.35, and 353.65 for pigs, cattle, and humans, respectively. The number of distinct eGenes and eVariants used in S-PrediXcan, FUSION, and UTMOST are presented in Tables S4 and S5. Figure S1 illustrates the number of overlapping eGenes and eVariants identified across the three software tools. The average of the estimated *cis*-heritability of the genes and the prediction performance of the models [the square of Pearson correlation (*R*^2^) between the predicted and observed expression in the five-fold cross-validation] are presented in Tables S6–S8. Figures S2 and S3 highlight the superior accuracy of the Elastic Net model in humans, whereas **Figure 2** demonstrates that BLUP and BSLMM models exhibit a higher accuracy than Elastic Net and CTIMP models in pigs and cattle. Figure S4 details the impact of varying sample sizes on model accuracy, with BLUP and BSLMM consistently outperforming other models across tissues.

A total of 38,180, 21,037, and 17,942 distinct eGenes are provided for humans, pigs, and cattle, respectively. They represent 13,780, 13,444, and 13,442 one-to-one orthologous genes in humans *vs*. pigs, humans *vs*. cattle, and cattle *vs*. pigs, respectively. The comparison of eGenes between species in terms of *cis*-heritability is presented in Tables S9–S11. The correlation of heritability estimates ranged from −0.1060 to 0.2300 across tissues when comparing humans and pigs. Among these tissues, the highest correlation (Pearson *r* = 0.2300, *P* = 1.94E−04) was observed for 258 shared orthologous genes tested between the human Brain_Anterior_cingulate_cortex_BA24 (*n* = 176) and pig hypothalamus (*n* = 74), followed by the correlation between the human left ventricle (*n* = 432) and pig heart (*n* = 165) (Pearson *r* = 0.2041, *P* = 9.35E−04). A heritability correlation of 0.1377 (*P* = 9.98E−07) was found for 1252 orthologous genes examined in human skeletal muscle (*n* = 803) and pig muscle (*n* = 1322) (Table S9). In contrast, the correlation of heritability estimates ranged from −0.0071 to 0.0733 when comparing humans and cattle (Table S10).

**Figure 3:**
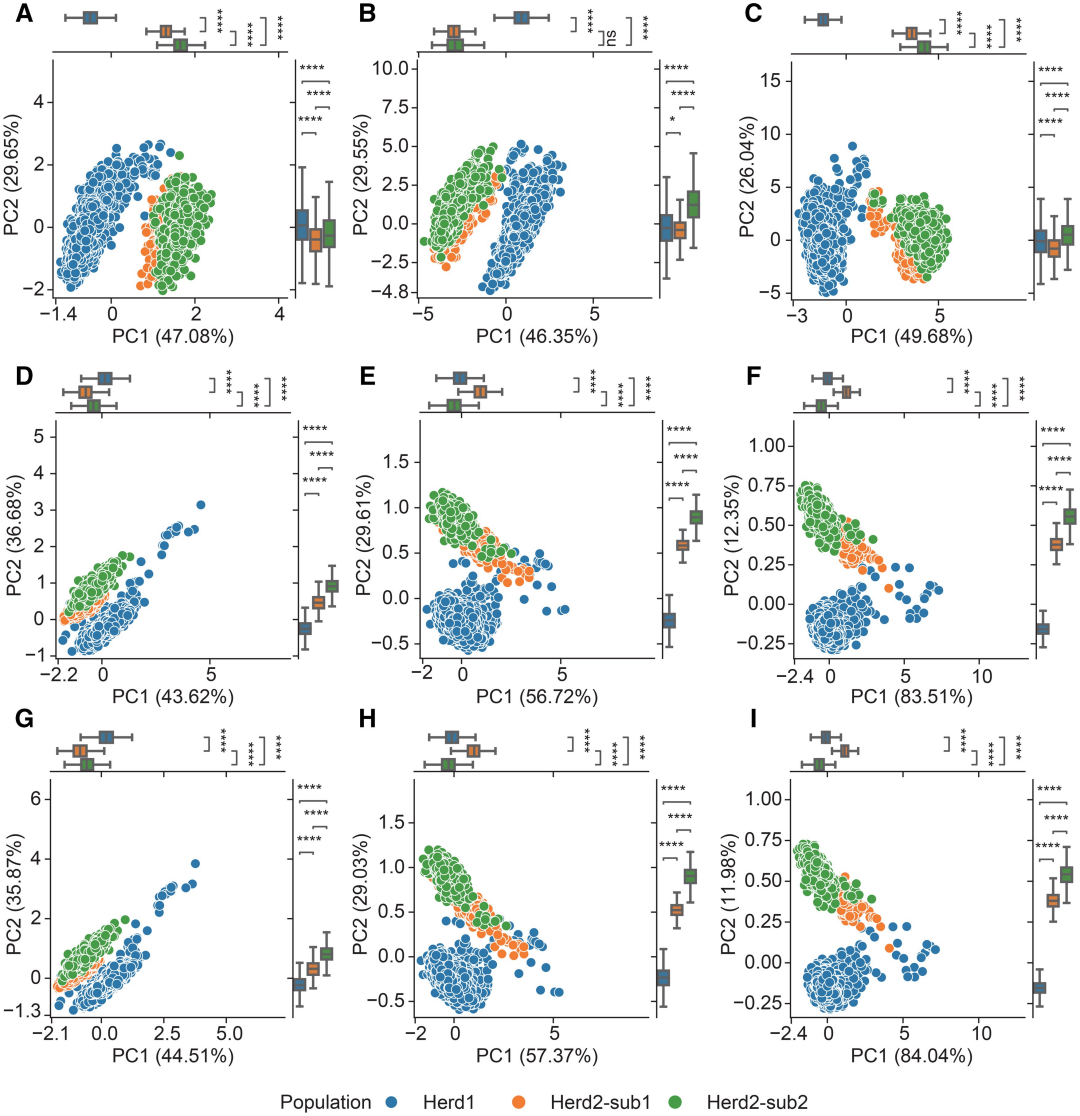
PCA analyses utilizing predicted expression levels **A**.–**C**. PCA results based on eQTLs trained by the Elastic Net model in the hypothalamus (A), blood (B), and muscle (C) tissues, respectively. **D**.–**F**. PCA results based on eQTLs trained by the BLUP model in the hypothalamus (D), blood (E), and muscle (F) tissues, respectively. **G**.–**I**. PCA results based on eQTLs trained by the BSLMM model in the hypothalamus (G), blood (H), and muscle (I) tissues, respectively. In all plots, blue dots represent pigs from Herd1, and orange and green dots indicate pigs from Herd2, which are from different locations. The box plots associated with each PCA plot display the variance explained by the respective PCs. The differences between PC values of different breeds were determined by *t*-test (*, *P* < 0.05; ****, *P* < 0.0001; ns, no significance). PCA, principal component analysis; PC, principal component; eQTL, expression quantitative trait locus.

We also implemented a module capable of computing predicted expression levels based on individual genotype data. As an illustration, we employed a dataset comprising 5445 pigs from three Duroc populations: Herd1, Herd2-sub1, and Herd2-sub2 (with Herd2-sub1 and Herd2-sub2 being sub-farms of Herd2). These pigs were genotyped using either a genotyping chip or whole-genome sequencing (WGS), and their genotypes were imputed to the PigGTEx level. Utilizing the TWAS-server, we generated predicted gene expression levels and performed principal component analysis (PCA). **Figure 3** shows that Elastic Net, BLUP, and BSLMM can effectively discern population structures. Notably, the Herd1 population exhibits a more clustered pattern in both BLUP and BSLMM, with principal component 1 (PC1) in these models accounting for a larger proportion of the explained variance.

### GWAS imputation module

**Figure 4:**
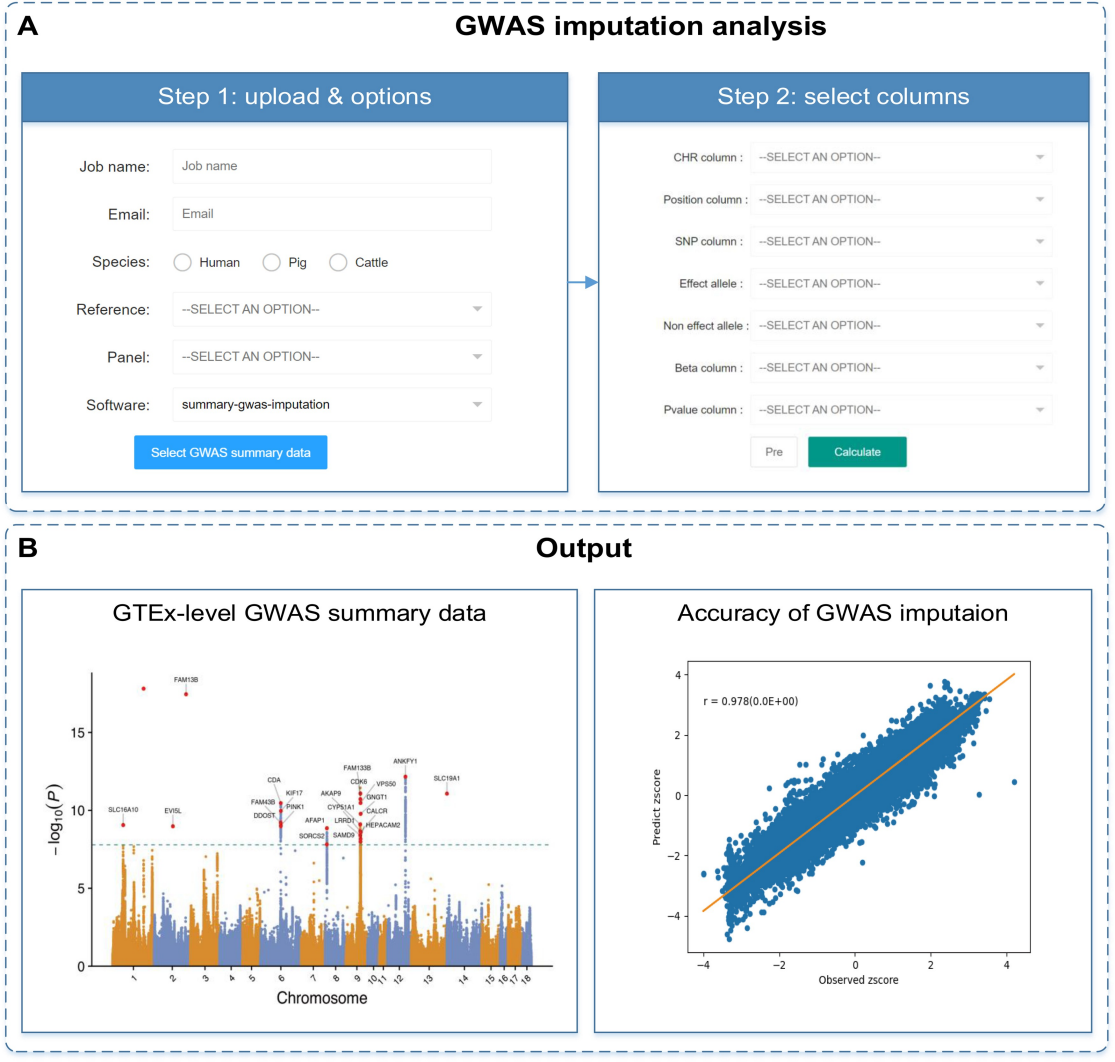
The operation flow for the “GWAS imputation” module **A**. Step 1: upload the GWAS summary statistics and select relevant options, such as species and genome assembly version. Step 2: assign appropriate column names for the uploaded data to ensure correct processing. **B**. An example output for the imputed GWAS summary statistics and imputation accuracy by five-fold cross-validation.

The “GWAS imputation” function is provided for imputing the GWAS summary statistics to the GTEx sequence level [*i.e.*, matching single nucleotide polymorphisms (SNPs) in the eQTL mapping reference population] according to the genotype imputation reference panel from the GTEx projects. This improves the power of TWAS analyses, particularly in farm animals where GWAS analyses are often conducted using low-density or high-density SNP arrays. The WGS data from PigGTEx [22] comprising 1602 samples with 42,523,218 variants [pig genomics reference panel (PGRP)] constitute the pig genotype imputation panel. The RNA sequencing (RNA-seq) data from CattleGTEx [21] were used to generate the 7394 samples with 3,824,445 variants that constitute the cattle genotype imputation panel. Additionally, the WGS data from animal genotype imputation database (AGIDB) [33,34], comprising 983 individuals with 12,842,237 single nucleotide variants (SNVs) after filtering, and from bovine genome variation database (BGVD) [35,36], comprising 2976 individuals with 17,149,833 SNVs after filtering, were collected as the reference panels for genotype imputation in cattle. The human genotype imputation reference panel comprises 500 Europeans with 27,731,499 variants. Users can perform harmonization, format standardization, missing data imputation, five-fold cross-validation, and result visualization in the GWAS imputation module. The “GWAS imputation” module consists of two essential steps. In Step 1, users provide their email address, which serves as the recipient for the result link sent by the server (as depicted in **Figure 4**A). Additionally, users select the species and genome assembly version. If the genome reference version differs from those used in FarmGTEx or GTEx (such as GRCh38/hg38, Sscrofa11.1/susScr11, or ARS-UCD1.2/bosTau9), the server will automatically perform a LiftOver to align the data. Users also have the option to choose between two imputation software: summary-gwas-imputation [37] and direct imputation of summary statistics (DIST) [38]. Moving on to Step 2, users upload their files to the server in a compressed format (.gz), and the server extracts the file header. In this step, users must assign names to each column, ensuring proper data organization and interpretation (Figure 4A). The server provides downloadable publication-quality figures (Figure 4B) along with text results. Furthermore, the five-fold cross-validation based on the longest chromosome will be used to output the imputation accuracy of the GWAS summary statistics (Figure 4B).

In this study, we evaluated the imputation accuracy using our GWAS data through ten replicates of a five-fold cross-validation approach. In each iteration, one-fifth of chromosome 1 was masked, ensuring no overlap between iterations. Pearson correlation coefficient was then calculated between the masked z-scores and the imputed z-scores. The imputation accuracy was approximately 80% for cattle milk traits and ranged from 80% to 90% for pig production traits (Figure S5).

### Online TWAS module

**Figure 5:**
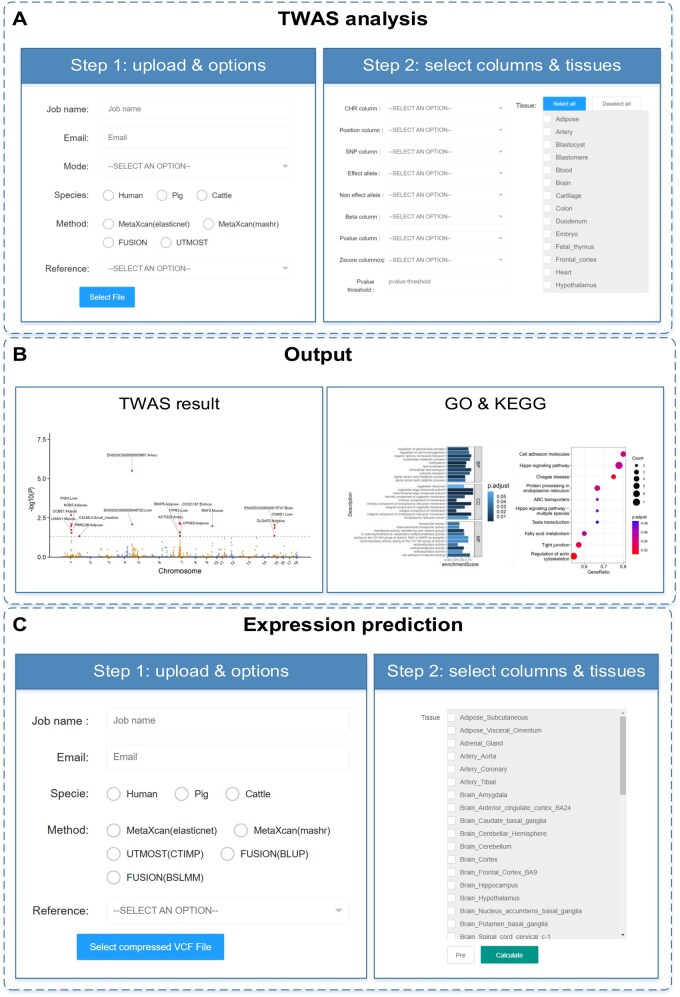
The operation flows for the “TWAS analysis” and “Expression prediction” modules **A**. The operation flow for the “TWAS analysis” module. Step 1: upload the GWAS summary statistics and select options, including species and imputation methods. Step 2: select the corresponding column names and tissues used for the TWAS analysis. **B**. The output of the “TWAS analysis” module includes (1) a Manhattan plot combining all TWAS results from multiple tissues, and (2) GO and KEGG gene enrichment analysis results visualized for the gene set enrichment. **C**. The operation flow for the “Expression prediction” module, where users can predict gene expression levels using individual genotype data. GO, Gene Ontology; KEGG, Kyoto Encyclopedia of Genes and Genomes.

The TWAS module is the central component of the FarmGTEx TWAS-server, enabling users to perform TWAS analysis across species (humans, pigs, and cattle) by uploading GWAS summary data. Additional livestock animals, such as chickens, sheep, and goats, will be included in future releases, as the FarmGTEx project is also working on these species. Users must upload the GWAS summary data file in Step 1 and select the column names in Step 2 (**Figure 5**A), similar to the GWAS imputation module. Users can select whether to perform GWAS imputation under “Mode” in Step 1. It will impute the genetic variants involved in the gene expression prediction models (Table S5). Users can choose from various software packages, such as MetaXcan (S-PrediXcan) [5], FUSION [6], and UTMOST [7], to perform TWAS analysis. To enhance the performance of FUSION, we modified the code and allowed it to run TWAS in parallel. For UTMOST, we first used S-PrediXcan for the single-tissue TWAS with the CTIMP prediction model, following which we performed a joint Generalized Berk-Jones test for all the TWAS summary statistics. Users can select multiple tissues for the TWAS analysis (up to 49, 34, and 23 tissues for humans, pigs, and cattle, respectively) in Step 2 and specify the cut-off *P* value (default is 0.05), with statistical significance set at 0.05/*n* (where *n* is the number of genes being tested). The TWAS module will perform the following seven steps on submission: (1) quality control, (2) LiftOver, (3) GWAS imputation, (4) TWAS analysis, (5) Manhattan plot illustration, (6) GSEA for Gene Ontology (GO) and Kyoto Encyclopedia of Genes and Genomes (KEGG) enrichment analyses of genes, and (7) result visualization. Upon completion of Step 3, a link documenting all the processes and results will be sent via email. Finally, five types of Manhattan plots can be downloaded directly, including (1) figures for GWAS, (2) figures for GWAS imputation, (3) figures for the *P* values and z-scores of the TWAS result per tissue, (4) figures for the *P* values of the TWAS results from all tissues (Figure 5B), and (5) an interactive post-Manhattan plot tab. The “Expression prediction” module (Figure 5C) allows users to predict gene expression based on the individual-level genotype data in addition to GWAS summary statistics for TWAS analysis. To perform this, users should upload the individual genotypes in variant call format (VCF, compressed in .gz), and the server will use PrediXcan to predict gene expression for each individual across tissues [5]. User data are stored on the server for one week and then deleted.

Moreover, we compared TWAS results between FarmGTEx TWAS-server and TWAS-hub based on a GWAS summary provided in TWAS-hub (29892013-GCST90029014-EFO_0006527.h.tsv.gz) [39]. TWAS-hub conducted the TWAS analysis using FUSION software with GTEx v6 data, while FarmGTEx TWAS-server utilized GTEx v8 data. As demonstrated in Figure S6, high correlations (Pearson *r* ≈ 90%) were observed for z-scores of the same genes across tissues.

### Search module

The FarmGTEx TWAS-server has curated TWAS results for 1129 distinct human traits and diseases using 2268 GWAS datasets. The TWAS summary data comprise 479,203 significant disease–tissue–gene trios (*P* < 0.05/*n*, *n* > 10). A total of 41 and 11 TWAS summary statistics for complex traits were incorporated for cattle [40] and pigs [41], respectively. Users can search TWAS results by gene query (Figure S7A). TWAS summary statistics for the queried gene, including Ensembl ID, gene symbol, trait, tissue, *P* value, and z-score, will be displayed (Figure S7B). The platform also provides detailed information about the queried gene and its orthologs in other species (Figure S7C), including gene location, homology type, and ortholog confidence. Additionally, the number of diseases/traits in the other species linked to the queried gene will be displayed (Figure S7D), and TWAS summary statistics can be accessed by clicking the hyperlink. Furthermore, it will present the gene expression profiles of orthologous genes across tissues in all available species (Figure S7E). Moreover, the TWAS results can also be searched on the web by a specific disease/trait, as shown in Figure S8A. Detailed information about the disease/trait, including disease/trait name, sample size, population, publication information, source links, and the number of associated tissue genes detected by S-PrediXcan, will also be provided (Figure S8B). The details of the TWAS results, including Ensembl ID, gene symbol, tissue, *P* value, z-score, and the number of associated genes in each of the tissues, can be accessed through hyperlink buttons (Figure S8C and D). In summary, users can explore the molecular mechanisms associated with a gene or a trait based on the large-scale TWAS results, which will be a valuable resource for translating genetic findings across species.

### Cross-species mapping module

The server includes an “Orthologous” module which enables users to upload the TWAS summary statistics from various species and perform orthologous gene comparisons. The comparison can be done by choosing the desired species and tissues (Figure S9A). The Pearson correlation coefficient will then be determined based on the *P* values and z-scores of one-to-one orthologous genes between species. These findings will help improve understanding of the evolutionary roots of a specific disease/trait and aid in the translation of genetic findings across species (Figure S9B and C).

### Comparison with existing online TWAS servers

The FarmGTEx TWAS-server is currently the only web platform supporting TWAS analysis across multiple species, including pigs, cattle, and humans. It provides a comprehensive module with several advantages over webTWAS [18] and TWAS hub [19]. First, unlike webTWAS and TWAS hub which are limited to human data, the FarmGTEx TWAS-server provides TWAS analysis and summary statistics across humans, cattle, and pigs. Additionally, future releases will include more farm animal species, such as chickens, goats, and sheep, in line with the FarmGTEx project. Beyond gene expression, upcoming updates will incorporate alternative molecular data types, including alternative splicing, promoter usage, and enhancer expression. Second, the FarmGTEx TWAS-server implements the multi-tissue TWAS method UTMOST, offering broader analytical capabilities compared to TWAS hub, which only supports single-tissue TWAS methods like FUSION. Similarly, webTWAS (http://www.webtwas.net/#/twas) provides analyses limited to S-PrediXcan and FUSION. Third, unlike TWAS hub and webTWAS which lack functional gene annotations, the FarmGTEx TWAS-server includes GSEA, enabling users to explore biological pathways and uncover significant functional insights within their data. Finally, the FarmGTEx TWAS-server provides additional visualization tools for uploaded and imputed GWAS summary statistics, including not only Manhattan plots of TWAS results but also illustrations that aid researchers in effectively interpreting and analyzing their data.

### Case studies

Herein, we present two case studies to demonstrate how the FarmGTEx TWAS-server aids researchers in performing TWAS analyses and uncovering the molecular mechanisms underlying complex traits across species.

#### Case study 1

**Figure 6:**
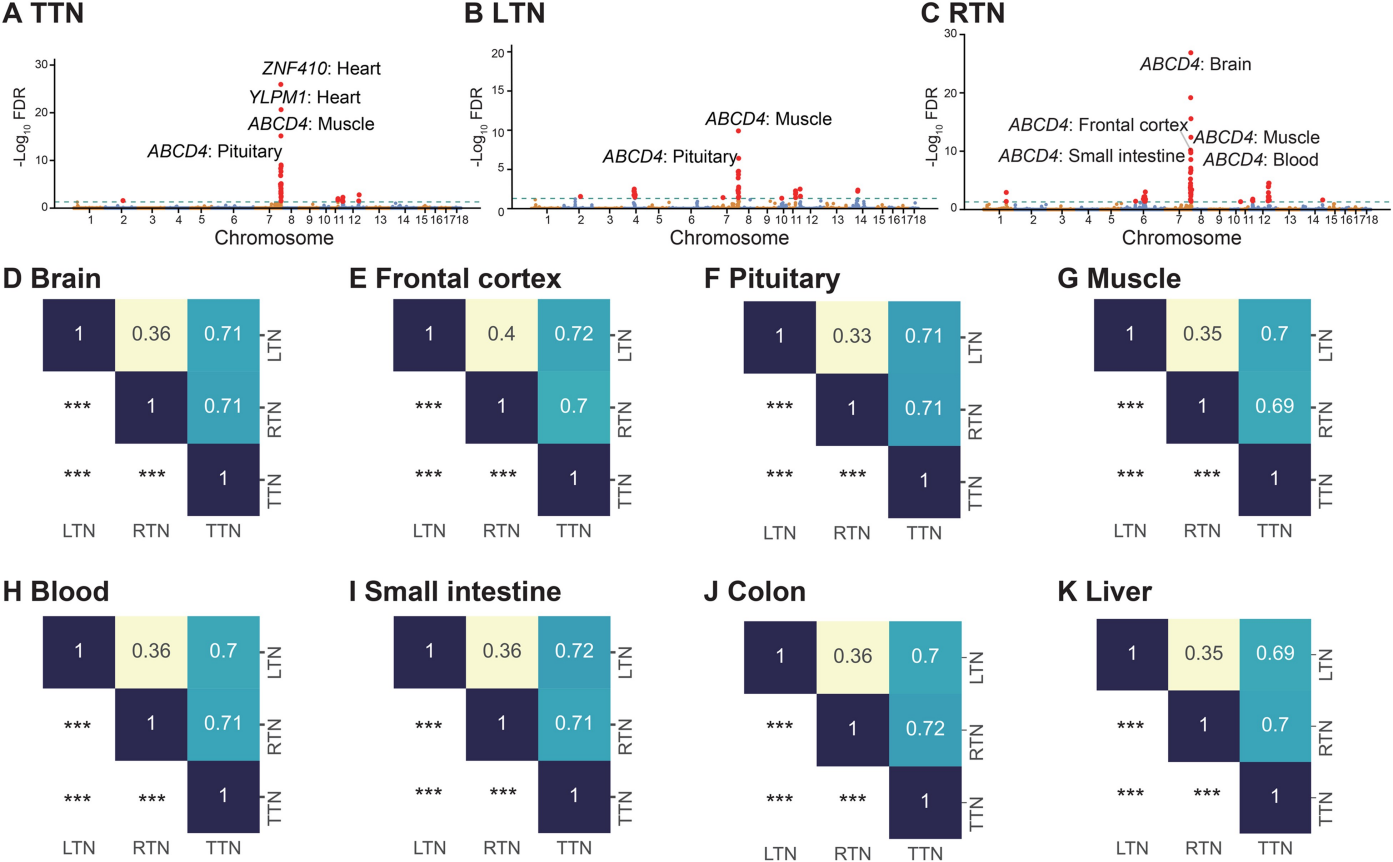
TWAS analysis of teat number using published GWAS summary statistics and Pearson correlation analysis of TWAS ***P*** values across various tissues **A**.–**C**. Manhattan plots displaying TWAS results for TTN (A), LTN (B), and RTN (C), with FDR values indicated. **D**.–**K**. Heatmaps showing the Pearson correlation coefficients of TWAS *P* values across traits in different tissues: brain (D), frontal cortex (E), pituitary (F), muscle (G), blood (H), small intestine (I), colon (J), and liver (K). The upper triangle of each heatmap presents the Pearson correlation coefficients, while the lower triangle shows the statistical significance of the Pearson correlations (***, *P* < 0.001). TTN, total teat number; LTN, left teat number; RTN, right teat number; FDR, false discovery rate.

We obtained GWAS summary statistics for total teat number (TTN) from a previous study [41], which identified a significant GWAS locus near the ATP binding cassette subfamily D member 4 (*ABCD4*) gene. Using the FarmGTEx TWAS-server, we performed a TWAS analysis that revealed significant associations between *ABCD4* expression in muscle and pituitary tissues and TTN (**Figure 6**A). Additionally, we found significant associations between *ABCD4* and left teat number (LTN) in muscle and pituitary tissues (Figure 6B), as well as right teat number (RTN) in the brain, frontal cortex, muscle, blood, and small intestine tissues (Figure 6C). We further examined the Pearson correlation coefficients of TWAS *P* values between various teat number traits across tissues (Figures 6D–K). The results showed that TTN was highly correlated with LTN (*r* = 0.69–0.72) and RTN (*r* = 0.69–0.72), while the correlation between RTN and LTN was lower (*r* = 0.33–0.4). These TWAS-based correlations were consistent with the phenotypic correlations reported by Yang et al. [41] (*r* = 0.82 between TTN and RTN, *r* = 0.83 between TTN and LTN, and *r* = 0.36 between RTN and LTN). Further verification of TWAS *P* values across tissues for teat number traits was conducted using another dataset [42]. We found that the Pearson correlation coefficients based on TWAS *P* values were 0.59–0.73 between LTN and RTN, 0.86–0.90 between LTN and TTN, and 0.86–0.90 between RTN and TTN (Table S12), which were consistent with the phenotypic correlations of 0.70, 0.93 and 0.91, respectively. These results suggested that the Pearson correlation coefficients based on the TWAS *P* values were similar to the phenotypic correlations for teat number traits from the previous dataset. This case study demonstrates that the FarmGTEx TWAS-server can not only provide regulatory mechanisms underlying GWAS loci by identifying associated genes in relevant tissues but also enhance the statistical power of association tests potentially by combining multiple signals of variants into a single gene.

#### Case study 2

We conducted a cross-ancestry meta-GWAS analysis for backfat thickness (BFT) using METASOFT [43] software. The dataset consisted of 2797 pigs from Yang et al. [41], 4045 pigs from figshare (https://figshare.com/articles/dataset/pig_data_and_simulated_phenotype/21130672), and 10,874 pigs genotyped by ourselves using low-coverage genome sequencing. In total, this dataset comprised 9369 Duroc pigs, 1854 Landrace pigs, 575 Pretrain pigs, and 5918 Yorkshire pigs. WGS data and chip data were imputed separately by GLIMPSE (v2) [44] and Beagle (v5.4) [45] using the PGRP reference panel. Subsequently, the imputed data were filtered based on the criteria of INFO score > 0.4 or DR2 > 0.4 and minor allele frequency (MAF) > 0.02. GWAS analyses were performed within the breed in each study using genome-wide efficient mixed-model association (GEMMA) [46], incorporating provided covariates and three principal components (PCs) as additional covariates. Meta-analyses were subsequently conducted using fixed-effect models via METASOFT software. As METASOFT exclusively considers signals shared across all studies, the final meta-GWAS summary statistics encompassed 14,858,204 variants.

**Figure 7:**
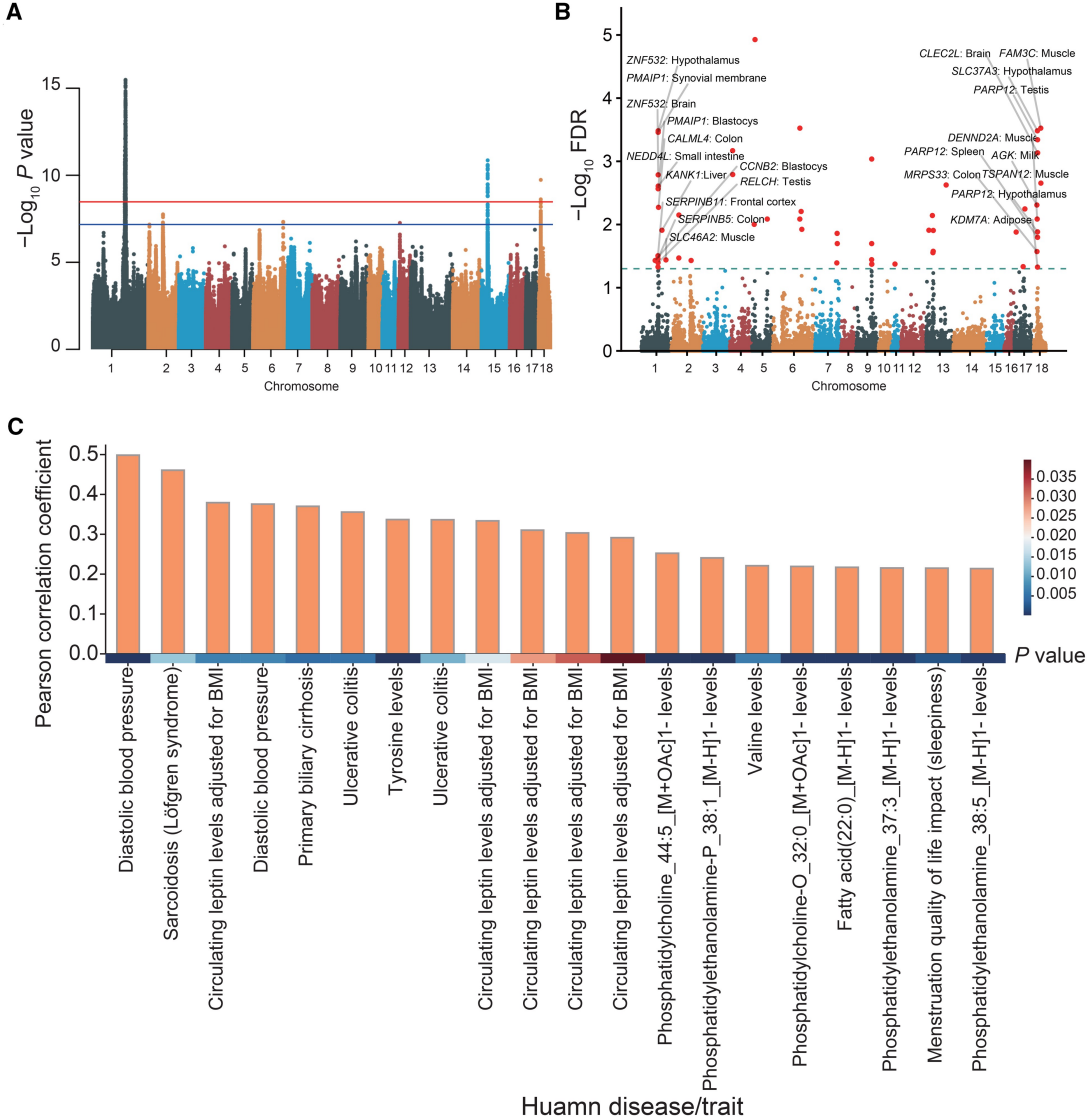
TWAS analysis based on meta-GWAS **A**. Manhattan plot depicting the meta-GWAS results. **B**. Manhattan plot illustrating the TWAS outcomes. **C**. Bar plot showing the Pearson correlation coefficients between pig backfat thickness and human diseases/traits based on TWAS summary statistics, with only significant results (*P* < 0.05) shown.

**Figure 7**A shows significant peaks in genomic regions SSC1: 157.9–161.9 Mb, SSC15: 30.66–30.76 Mb, and SSC18: 10.07–10.12 Mb, with *P* < 3.36E−09 (Bonferonni correction). To complement these findings, we performed the TWAS analysis using the server with FUSION software. The Manhattan plot in Figure 7B depicts associations across the genome in different tissues. Notably, *ZNF532* (in hypothalamus and brain), *PMAIP1* (in synovial membrane and blastocyst), *NEDD4L* (in small intestine), *CALML4* (in colon), *SERPINB11* (in frontal cortex), *SERPINB5* (in colon), *CCNB2* (in blastocyst), *RELCH* (in testis), and ENSSSCG00000046336 (in large intestine) were identified to be associated with BFT around SSC1: 158.5–166.1 Mb. Similarly, in SSC18, near the significantly associated GWAS signals, *SLC37A3* (in hypothalamus), *PARP12* (in testis, hypothalamus, and spleen), *CLEC2L* (in brain), *DENND2A* (in muscle), *MRPS33* (in colon), and *KDM7A* (in adipose) were identified to be associated with BFT around SSC18: 8.88–10.16 Mb. Remarkably, the TWAS findings were aligned closely with the GWAS results. *PMAIP1* has been reported to be associated with obesity, body mass index (BMI), and height in humans [47] and average daily gain (ADG) in pigs [48], while *DENND2A* has been proposed as a candidate gene for BFT [49].

Moreover, when comparing TWAS summary statistics for humans using the Orthologous module, we uncovered stronger associations between pig BFT and human diseases/traits including diastolic blood pressure, sarcoidosis (Löfgren syndrome), and BMI (Figure 7C). These cross-species correlations further support our findings.

## Discussion and future prospects

In this study, we introduced the FarmGTEx TWAS-server, an interactive web resource designed for customized TWAS analyses and functional annotations across multiple tissues and species (humans, cattle, and pigs). Researchers can upload individual genotypes and GWAS summary statistics to generate TWAS results or query pre-existing TWAS data by gene or trait. The server provides an effective platform for mapping genes associated with complex traits and enables cross-species translation of genetic findings, as demonstrated in our case studies.

The FarmGTEx TWAS-server currently supports three species and provides gene expression prediction models across a broad spectrum of tissues. As the FarmGTEx project progresses, we plan to expand the server to include additional tissues, cell types, molecular phenotypes (*e.g.*, alternative splicing and enhancer expression), and species. Furthermore, we are committed to ongoing updates to the software and models, including the integration of advanced GWAS imputation tools to improve computational speed and accuracy, the implementation of new models for eQTL analysis, and the incorporation of the latest TWAS methodologies. This resource will continue to be invaluable for the scientific community in exploring genetic mechanisms and translating genetic insights across species and breeds.

## Materials and methods

### Gene expression data collection and normalization

We collected the expression data [transcripts per million (TPM)] of 26,908 and 27,537 genes from 34 and 23 tissues of pigs and cattle from the FarmGTEx project [21,22], respectively. Details of these samples are summarized in Tables S1 and S2. Genes with TPM < 0.1 and raw read count < 6 in more than 20% of samples were excluded for each tissue in pigs and cattle, resulting in 5457 pig samples and 4889 cattle samples for downstream analysis. The trimmed mean of M (TMM) values [50], followed by the inverse normal transformation of TMM values, were used for sample-wise correction of the gene expression values [21,22]. Human gene expression data were obtained from the GTEx database (https://www.gtexportal.org/), covering 55,878 genes from 54 human tissues [11], among which five tissues, including bladder (*n* = 21), cervix_ectocervix (*n* = 9), cervix_endocervix (*n* = 10), fallopian_tube (*n* = 9), and kidney_medulla (*n* = 4), were excluded owing to the small sample size. A summary of sample sizes for human is available in Table S3.

### Gene expression prediction model training

Multiple prediction models were used to impute gene expression, including Elastic Net model in S-PrediXcan [5], TOP1, BLUP, and BSLMM models in FUSION [6], and CTIMP in UTMOST [7]. For humans, the prediction models were downloaded from https://zenodo.org/record/3519321/ (Elastic Net model), http://gusevlab.org/projects/fusion/ (FUSION), and https://zenodo.org/record/3842289 (UTMOST). For pigs and cattle, the Elastic Net models were obtained from https://www.farmgtex.org/; FUSION (TOP1, BLUP, and BSLMM) and UTMOST (CTIMP) were used to construct the prediction models for pigs and cattle following the same pipeline as used for human data [6,7], with detailed parameters described in previous studies [21,22]. Ten probabilistic estimation of expression residuals (PEER) factors were estimated using PEER (v1.3) [51] based on the gene expression matrix to account for hidden batch effects of transcriptome-wide variation in gene expression within each tissue. Genotype PCs were estimated using PLINK (v1.9) [52] based on the genotype data to account for the population structure. The number of genotype PCs included was based on the sample size, which was as follows: 5 PCs for tissues with < 200 samples and 10 PCs for tissues with ≥ 200 samples. The *cis*-window of a gene was defined as 1 Mb upstream and downstream of its transcription start site. The prediction models of FUSION were then trained using the command: Rscript FUSION.compute_weights.R - -bfile $OUT  - -tmp $OUT.tmp  - -out $FINAL_OUT  - -verbose 0  - -save_hsq  - -PATH_gcta $GCTA  - -PATH_gemma $GEMMA --PATH_plink $PLINK2  - -models top1,blup,bslmm  - -covar $TISSUE.covariates4Fusion.txt  - -crossval 5. For CTIMP models, the command from https://github.com/yiminghu/CTIMP was followed.

### GWAS data collection and quality control

We collected GWAS summary statistics of 1129 human traits from the GWAS Catalog [53], webTWAS [18], and the Neale Lab UK Biobank (v3, http://www.nealelab.is/uk-biobank), 11 pig traits [41], and 41 cattle traits [40] to demonstrate the applicability of the TWAS-server. Besides, GWAS results of four pig traits, generated from newly acquired genotypes of 2778 Duroc pigs, were included. The GGP Porcine 50K v1 Genotyping BeadChip (Neogen GeneSeek, Lincoln, NE) (*n* = 974) or low-coverage WGS (depth = 1×, *n* = 1804) was used for genotyping the Duroc pigs. Beagle (v5.4) [45] was then used to impute missing genotypes with the current version of PGRP from the PigGTEx, comprising WGS data of 1602 pigs from over 100 breeds worldwide [22]. GWAS analysis for four traits was then performed using GEMMA [46], including birth weight (BW), corrected days to 115 kg (DAY115), BFT at 115 kg (BFT115), and loin muscle area at 115 kg (LMA115).

Only the GWAS summary statistics with complete information, including the rsID or variant coordinate, effect/non-effect allele, *P* value, beta coefficient, and z-score, were considered. The following quality control steps were performed to ensure that the GWAS data format was acceptable by TWAS. (1) For the human dataset, reference SNP cluster IDs were retrieved from dbSNP build 151 for variants only with variant coordinates. For animal datasets, variant coordinates based on Sscrofa11.1/susScr11 (pig) or ARS-UCD1.2/bosTau9 (cattle) were used. LiftOver analysis was performed using pyliftover (v0.4, https://pypi.org/project/pyliftover/) if the GWAS summary statistics were based on different genome assemblies. (2) GWAS summary statistics lacking either the non-effect alleles or effect alleles were excluded. (3) GWAS summary statistics without *P* values and beta coefficients were also removed. (4) Results with less than ten genes being tested after TWAS analysis were discarded.

### Imputation module for GWAS summary statistics

The imputation module for GWAS summary statistics was constructed to enhance the TWAS power. The genome reference panels were obtained from the 1000 Genomes Project [54], CattleGTEx [21], and PigGTEx [22] for humans, cattle, and pigs, respectively. The WGS panel included 27,731,499 variants (*n* = 500 samples) for humans, 3,824,445 variants (*n* = 7394 samples; obtained from RNA-seq data and subsequently imputed to WGS) for cattle, and 42,523,218 variants (*n* = 1602 samples) for pigs. The WGS data from AGIDB [33,34] encompassed 983 cattle with 20,211,4418 SNVs and were filtered to retain 12,842,237 variants with MAF > 0.05. Similarly, the WGS data from BGVD [35,36] included 2976 cattle with 58,466,684 SNVs, and 17,149,833 variants were retained after MAF filtering. The filtered WGS data were integrated as the cattle imputation reference panel. In online TWAS analysis, only SNPs identified as significant variants (eVariants) in the gene expression prediction model were considered for “GWAS imputation” to lower the computational burden. Table S5 presents the number of eVariants for each prediction model.

Two software packages were considered for GWAS summary statistics imputation: (1) the Python-based software package summary-gwas-imputation developed by Barbeira et al. [37] and (2) the C++-based DIST for unmeasured SNPs [38]. However, DIST did not initially support cattle GWAS imputation because it did not permit chromosomes with numbers greater than 22. Consequently, respective panels were constructed using the pipelines from summary-gwas-imputation (https://github.com/hakyimlab/summary-gwas-imputation) and DIST (https://github.com/dleelab/dist), as they use different formats of genome reference panels as input. Additionally, chromosomes with the largest number of SNPs were used to evaluate the imputation accuracy, specifically assessing the Pearson correlation coefficient between the imputed z-score and the observed z-score through a five-fold cross-validation approach.

### The workflow for online TWAS analysis

To provide a comprehensive and user-friendly TWAS web server, users can perform a range of functions, including quality control, LiftOver, GWAS summary imputation, TWAS analysis, and GSEA, by only uploading their GWAS summary statistics. All results, along with publication-quality figures, are available for download. The uploaded GWAS summary statistics file should comprise columns for chromosome, position, SNP name, effect allele, non-effect allele, *P* value, and beta coefficient. The server checks the reference assembly, SNP, and chromosome for quality control. pyliftover 0.4 (https://pypi.org/project/pyliftover/) will be used by the server to convert the genomic coordinates if the reference assembly of GWAS does not match that of GTEx or FarmGTEx. As previously detailed, GWAS imputation is applied to the data. Multiple software packages, including two single-tissue TWAS methods (*i.e.*, S-PrediXcan [5] and FUSION [6]) and a multi-tissue TWAS method (UTMOST [7]), can be selected for TWAS analysis, and GSEA analysis can be performed using clusterProfiler to explore the function of a gene list [55]. Upon job completion, users will receive an email containing a link to access all job processes and results. Moreover, the server provides an “Expression prediction” module, based on the PrediXcan [5] software package, for users with individual-level data. This module allows users to assess the associations between genetically regulated gene expression levels and phenotypes of interest using the imputed gene expression levels. A total of 2268, 41, and 11 TWAS summary statistics were provided based on S-PrediXcan [5] for humans, cattle, and pigs, respectively. A user-friendly interface was developed to facilitate the search and querying of results. For humans, TWAS analysis was conducted directly using the GWAS summary statistics due to the high density of SNPs in GWAS. For pigs and cattle, GWAS imputation was conducted prior to TWAS analysis. Significant disease/trait/tissue–gene associations are defined as genes with a *P* value below a threshold of 0.05/*n*, where *n* is the number of genes tested in a TWAS analysis.

### Database and TWAS web server

The back end of the TWAS-server was constructed using the Hypertext Preprocessor-based ThinkPHP5.0 web framework (https://www.thinkphp.cn/), and the front end was developed with the Layui framework (https://github.com/layui/layui) and jQuery JavaScript library (https://jquery.com/). The database was established based on MySQL. Python and R were used to develop the computational pipelines in the TWAS-server, and ggplot2 [56] in R [57] was used to visualize the data and results.

## Code availability

The code is available at https://github.com/ZhangZhenYang-zzy/TWAS. The code has also been submitted to BioCode at the National Genomics Data Center (NGDC), China National Center for Bioinformation (CNCB) (BioCode: BT007941), which is publicly accessible at https://ngdc.cncb.ac.cn/biocode/tools/BT007941.

## Supplementary Material

qzaf006_Supplementary_Data

## Data Availability

The FarmGTEx TWAS-server is publicly available at https://twas.farmgtex.org. It has also been submitted to Database Commons [58] at the NGDC, CNCB, which is publicly accessible at https://ngdc.cncb.ac.cn/databasecommons/database/id/9669.

## CRediT author statement

**Zhenyang Zhang:** Conceptualization, Data curation, Formal analysis, Investigation, Methodology, Software, Resources, Validation, Visualization, Writing – original draft, Writing – review & editing. **Zitao Chen:** Conceptualization, Data curation, Methodology, Software, Resources, Writing – review & editing. **Jinyan Teng:** Conceptualization, Data curation, Methodology, Resources, Writing – review & editing. **Shuli Liu:** Conceptualization, Data curation, Methodology, Resources, Writing – review & editing. **Qing Lin:** Data curation, Resources. **Jun Wu:** Data curation, Resources. **Yahui Gao:** Conceptualization, Data curation, Resources. **Zhonghao Bai:** Data curation, Resources. **Bingjie Li:** Conceptualization, Writing – review & editing, Funding acquisition. **George Liu:** Conceptualization, Writing – review & editing, Methodology, Supervision, Conceptualization. **Zhe Zhang:** Conceptualization, Writing – review & editing, Methodology, Supervision, Conceptualization. **Yuchun Pan:** Conceptualization, Funding acquisition, Writing – review & editing, Methodology, Supervision, Data curation, Resources, Validation. **Zhe Zhang:** Conceptualization, Writing – review & editing, Investigation, Methodology, Supervision, Data curation, Resources, Funding acquisition, Validation. **Lingzhao Fang:** Conceptualization, Writing – review & editing, Investigation, Methodology, Supervision, Data curation, Resources, Funding acquisition, Validation. **Qishan Wang:** Conceptualization, Writing – review & editing, Investigation, Methodology, Supervision, Data curation, Resources, Funding acquisition, Validation. All authors have read and approved the final manuscript.
