# Supplementary material for: FarmGTEx TWAS-server: An Interactive Web Server for Customized TWAS Analysis
Source: Genomics Proteomics Bioinformatics. 2025 Feb 11;23(1):qzaf006. doi: 10.1093/gpbjnl/qzaf006 (PMC12237508; doi:10.1093/gpbjnl/qzaf006)
Supplement: qzaf006_Supplementary_Data [file qzaf006_supplementary_data.zip › supplementary material captions.docx]

# Supplementary material

**Figure S1 Comparison among three software tools**

**A.–C.** The number of eGenes identified by different models and their intersections for pigs (A), cattle (B), and humans (C). **D.–F.** The intersections of variants for pigs (D), cattle (E), and humans (F). eGenes, the expressed genes with significant eQTL.

**Figure S2 Prediction performance in human tissues**

Box plots illustrating the prediction performance (R^2^) from five-fold cross-validation for different prediction models across human tissues. X-axis: human tissues. Y-axis: R^2^ values indicating the strength of correlation between predicted and observed expression levels. Box colors correspond to different models: CTIMP (blue), Elastic Net (orange), LASSO (green), TOP1 (red), and Elastic Net trained by FUSION (purple). LASSO, least absolute shrinkage and selection operator.

**Figure S3 The accuracy comparison of the various prediction models in human**

The bar plot illustrates the number of eGenes in various models. The scatter plot depicts a prediction performance comparison between Elastic Net and four other models (TOP1, LASSO, Elastic Net from FSUION, CTIMP from UTMOST) across three human tissues. **A.** Lung. **B.** Whole blood. **C.** Skeletal muscle. The X-axis and Y-axis represent the Pearson correlation between the predicted expression and observed expression in the five-fold cross-validation. The orange and other colored circles denote genes imputable using only Elastic Net and the other approaches, respectively. The black and grey dots denote genes consistently imputable and not imputable, respectively, using both methods.

**Figure S4**  **The accuracy comparison of the various prediction models in pigs and cattle**

The bar plot illustrates the number of eGenes in various models for pig and cattle. The scatter plot depicts a prediction performance comparison between Elastic Net and four other models (TOP1, BLUP, BSLMM, CTIMP from UTMOST) in three tissues for pigs and cattle. **A.** Hypothalamus in pigs. **B.** Blood in pigs. **C.** Muscle in pigs. **D.** Leukocyte in cattle. **E.** Mammary in cattle. **F.** Muscle in cattle. The X-axis and Y-axis represent the Pearson correlation between the predicted expression and observed expression in the five-fold cross-validation. The orange and other colored circles denote genes imputable using only Elastic Net and the other approaches, respectively. The black and grey dots denote genes consistently imputable and not imputable, respectively, using both methods.

**Figure S5 The accuracy of imputing GWAS summary statistics**

**A.** Pearson correlation between the z-scores and imputed z-scores for cattle milk traits. Fatpercent represents the fat percentage in milk, fatyield represents the fat yield, milkprotein represents the milk protein percentage, and milkyield represents the milk yield. Propercent refers to the protein percentage in milk. D5 and D305 indicate that the traits were recorded on the 5th and 305th days postpartum, respectively. **B.** Pearson correlation between the z-scores and imputed z-scores for pig production traits across various breeds.

**Figure S6 Scatter plot of TWAS z-scores from TWAS-hub and FarmGTEx TWAS-server**

**A.** TWAS z-scores were calculated using the Elastic Net model. **B.** z-scores were calculated using the LASSO model.

**Figure S7 Operation flow for “Search by gene****”**

**A.** Screenshot of the search box where users can select species and use autocompletion to choose the gene of interest. **B.** TWAS results of the target gene associated with traits in the selected species. **C.** Basic information of the target gene and its orthologs in two other species. **D.** The number of genes associated with the target gene across different species. **E.** Expression levels of the target gene across different tissues in the species.

**Figure S8 Screenshots of “Search by trait”**

**A.** Screenshot of the search box where users can select species and use autocompletion to choose the trait of interest. **B.** Overview of traits across different studies, providing specific source articles and the number of significantly associated tissue–gene pairs. Clicking the corresponding numbers will display detailed information. **C.** TWAS summary statistics for the trait presented in a tabular format. **D.** Bar chart showing the number of associated genes across different tissues.

**Figure S9 Screenshots of the “Orthologous module”**

**A.** Upload the GWAS summary statistics and select the options. **B.** A screenshot of the results, which includes information on traits such as publication date, journal, PubMed ID, sample size, and the correlation coefficients based on *P* values and z-scores for the uploaded file and the trait. **C.** A scatter plot showing the *P* values of the Pearson correlation coefficient. And a bar chart displaying the Pearson correlation coefficients.

**Table S1 The sample size of the RNA-seq samples for pig, and the number of the eVariants and eGenes of the eQTL models**

**Table S2 The sample size of the RNA-seq samples for cattle, and the number of the eVariants and eGenes of the eQTL models**

**Table S3 The sample size of the RNA-seq samples for human, and the number of the eVariants and eGenes of the three eQTL models**

**Table S4**  **The number of distinct eGenes identified by various methods in humans, pigs, and cattle**

**Table S5**  **The number of distinct significant variants in the gene expression prediction model identified by various methods in humans, pigs, and cattle**

**Table S6** **The average heritability in each tissue and the average cross-validation R^2^ in the prediction models for pigs**

**Table S7 The average heritability in each tissue and the average cross-validation R^2^ in the prediction models for cattle**

**Table S8 The average heritability in each tissue and the average cross-validation R^2^ in the prediction models for humans**

**Table S9** **The tissue pairs used in the comparative analysis between humans and pigs**

**Table S10** **The tissue pairs used in the comparative analysis between humans and cattle**

**Table S11** **The tissue pairs used in the comparative analysis between pigs and cattle**

**Table S12 The Pearson correlation coefficient based on TWAS *P* values were calculated for the correlation between LTN and RTN, LTN and TTN, and RTN and TTN**
