# Supplementary figures and images for: FarmGTEx TWAS-server: An Interactive Web Server for Customized TWAS Analysis
Source: Genomics Proteomics Bioinformatics. 2025 Feb 11;23(1):qzaf006. doi: 10.1093/gpbjnl/qzaf006 (PMC12237508; doi:10.1093/gpbjnl/qzaf006)

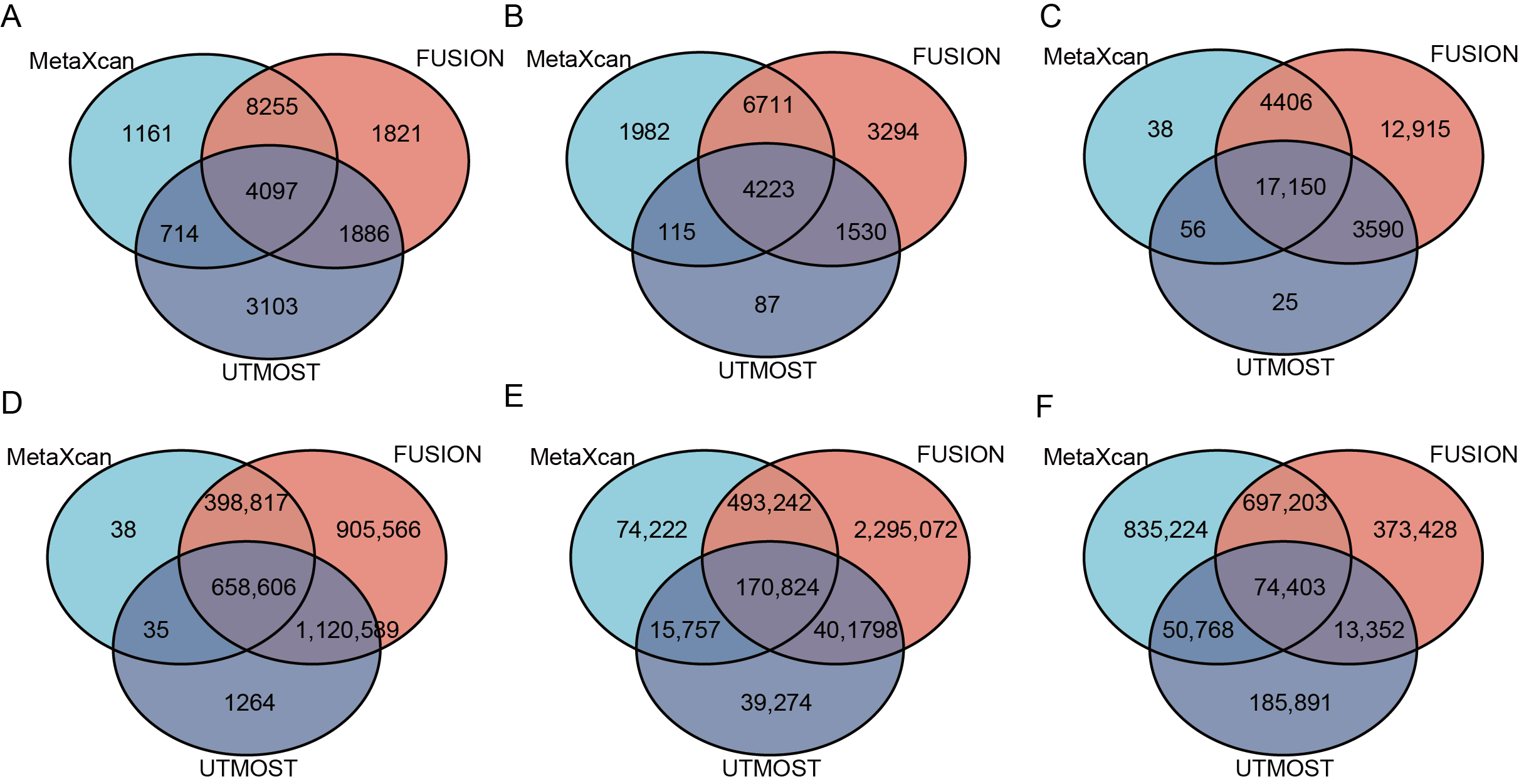

Supplement: qzaf006_Supplementary_Data [file qzaf006_supplementary_data.zip › Supplementary Figure 1.png]

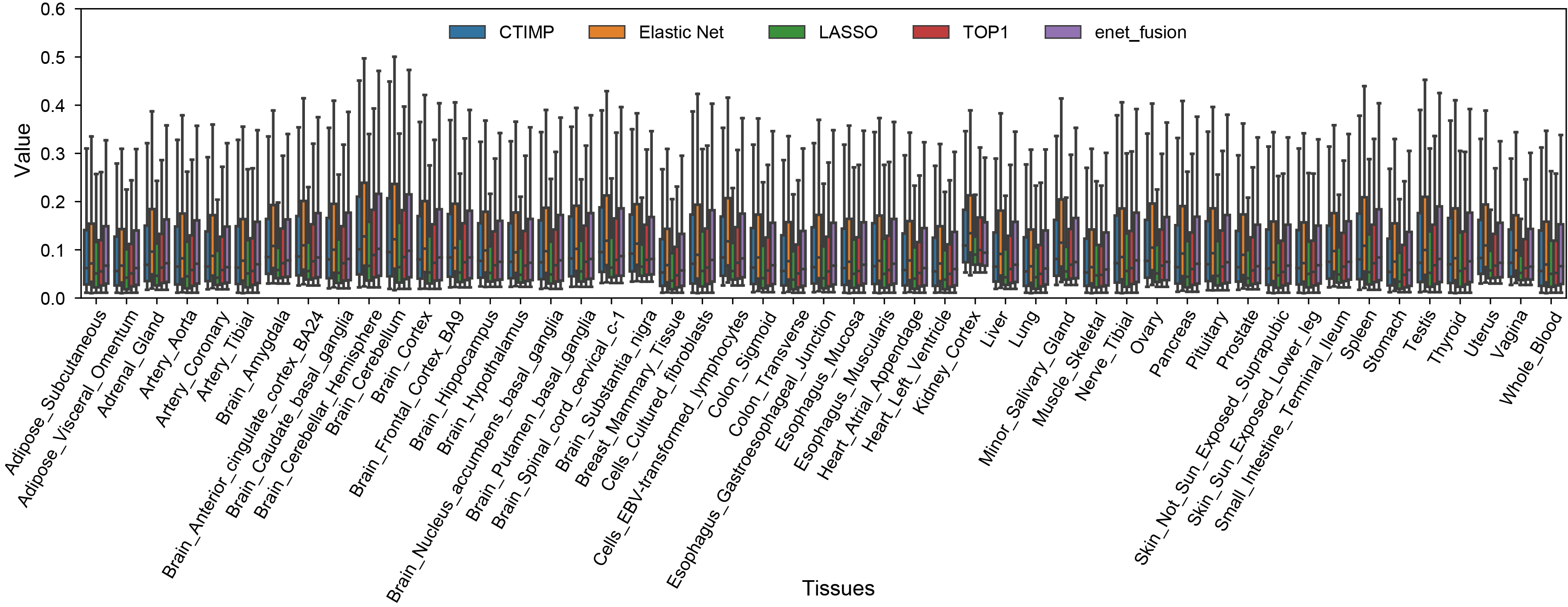

Supplement: qzaf006_Supplementary_Data [file qzaf006_supplementary_data.zip › Supplementary Figure 2.png]

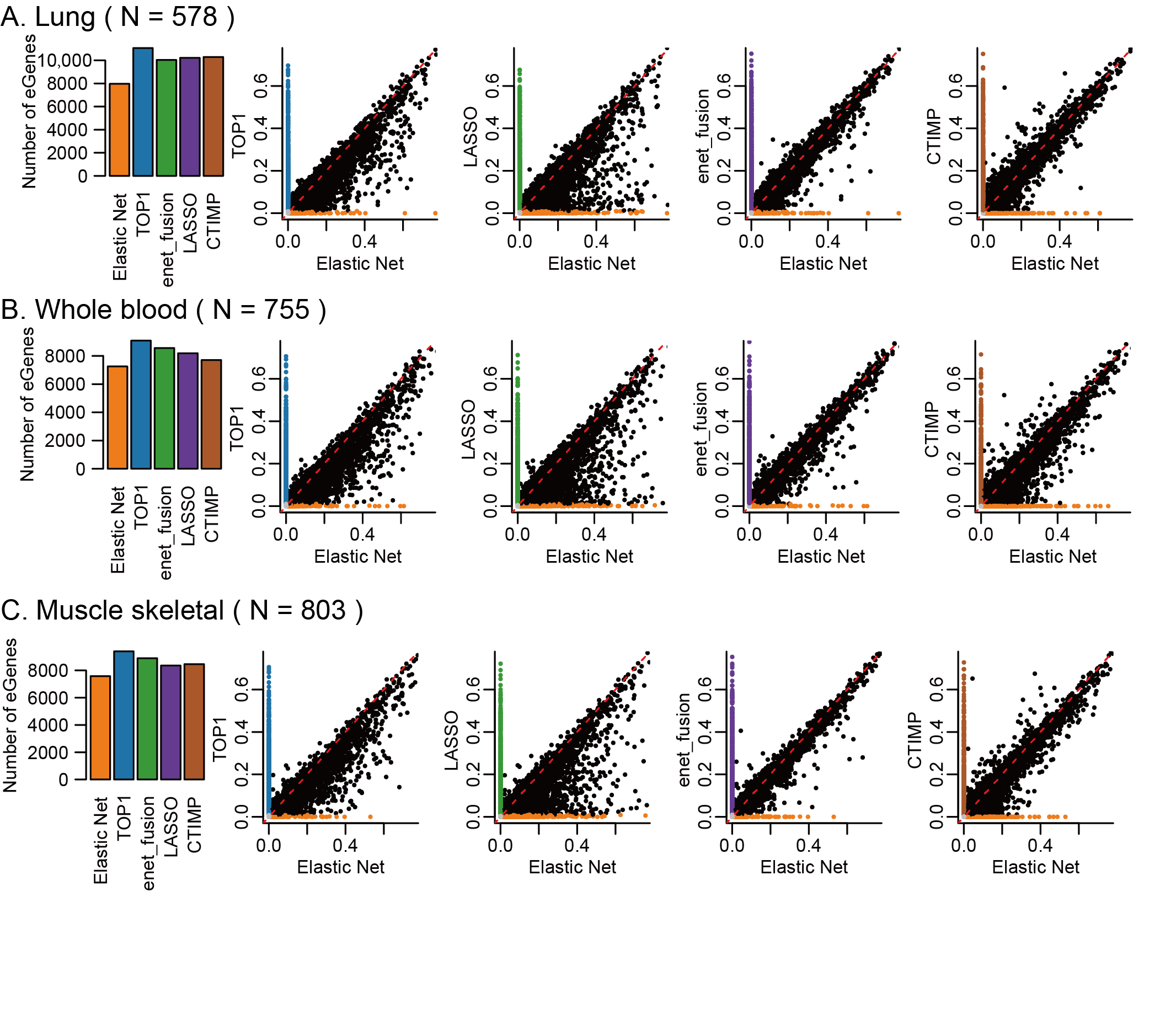

Supplement: qzaf006_Supplementary_Data [file qzaf006_supplementary_data.zip › Supplementary Figure 3.png]

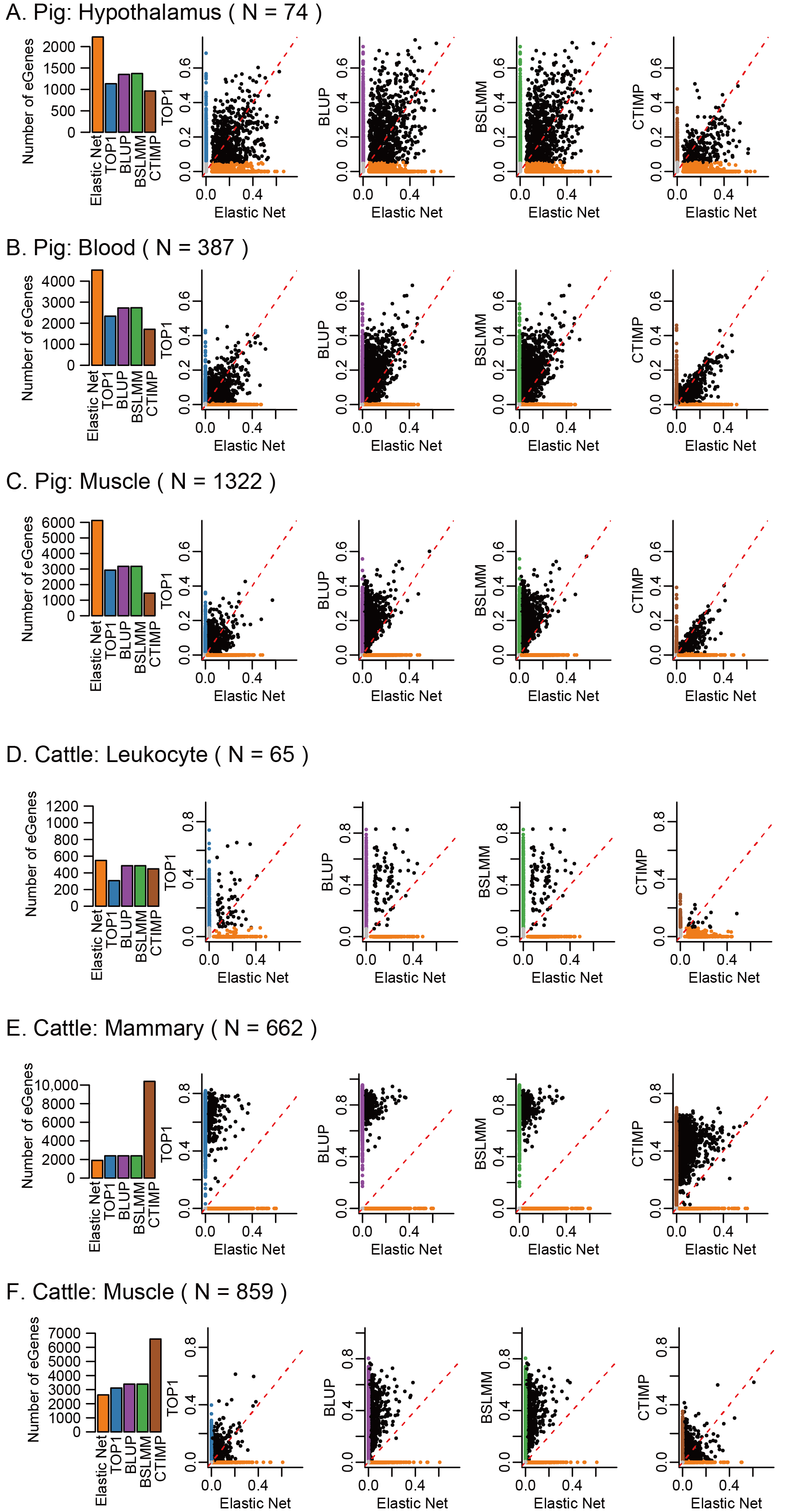

Supplement: qzaf006_Supplementary_Data [file qzaf006_supplementary_data.zip › Supplementary Figure 4.png]

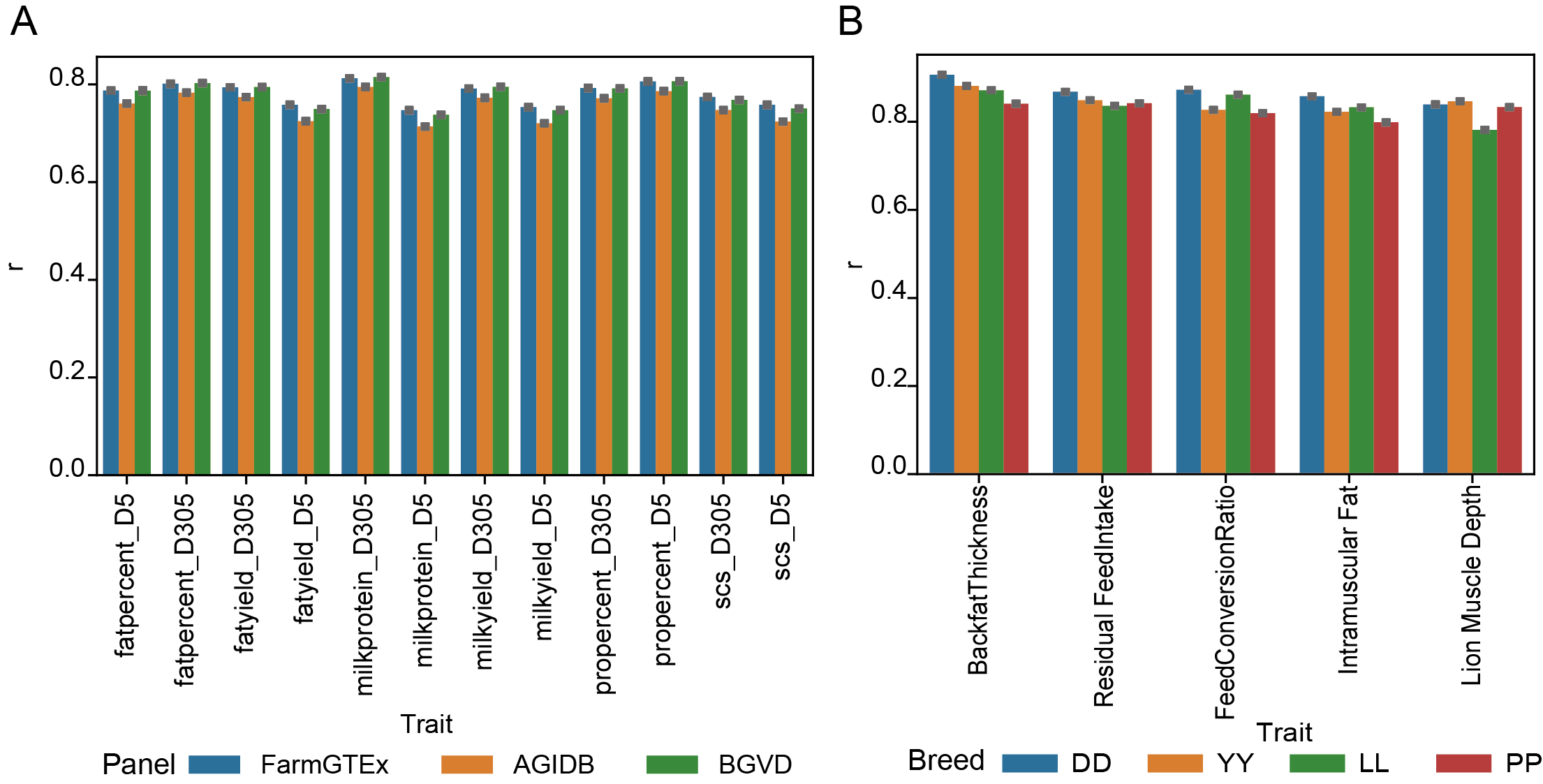

Supplement: qzaf006_Supplementary_Data [file qzaf006_supplementary_data.zip › Supplementary Figure 5.png]

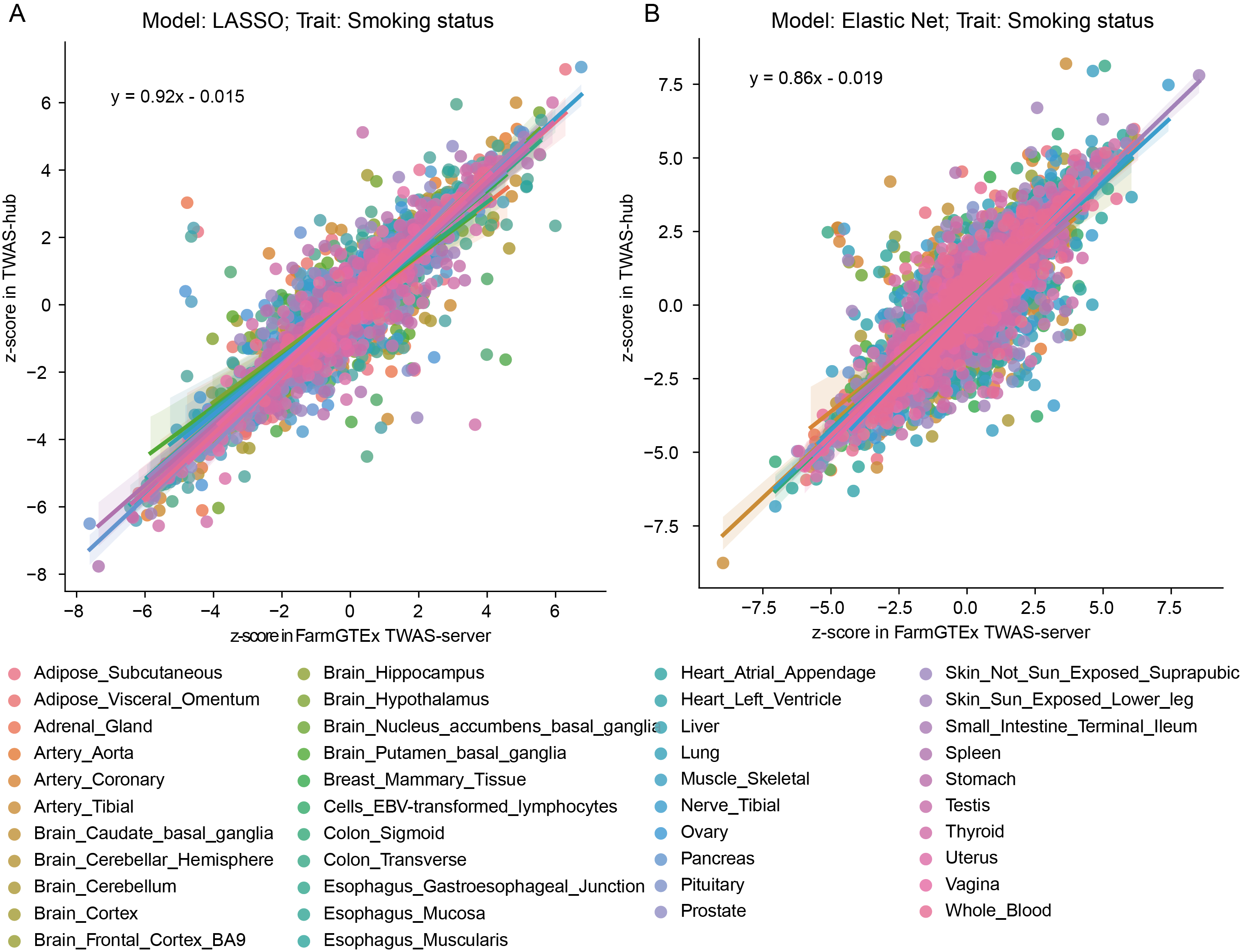

Supplement: qzaf006_Supplementary_Data [file qzaf006_supplementary_data.zip › Supplementary Figure 6.png]

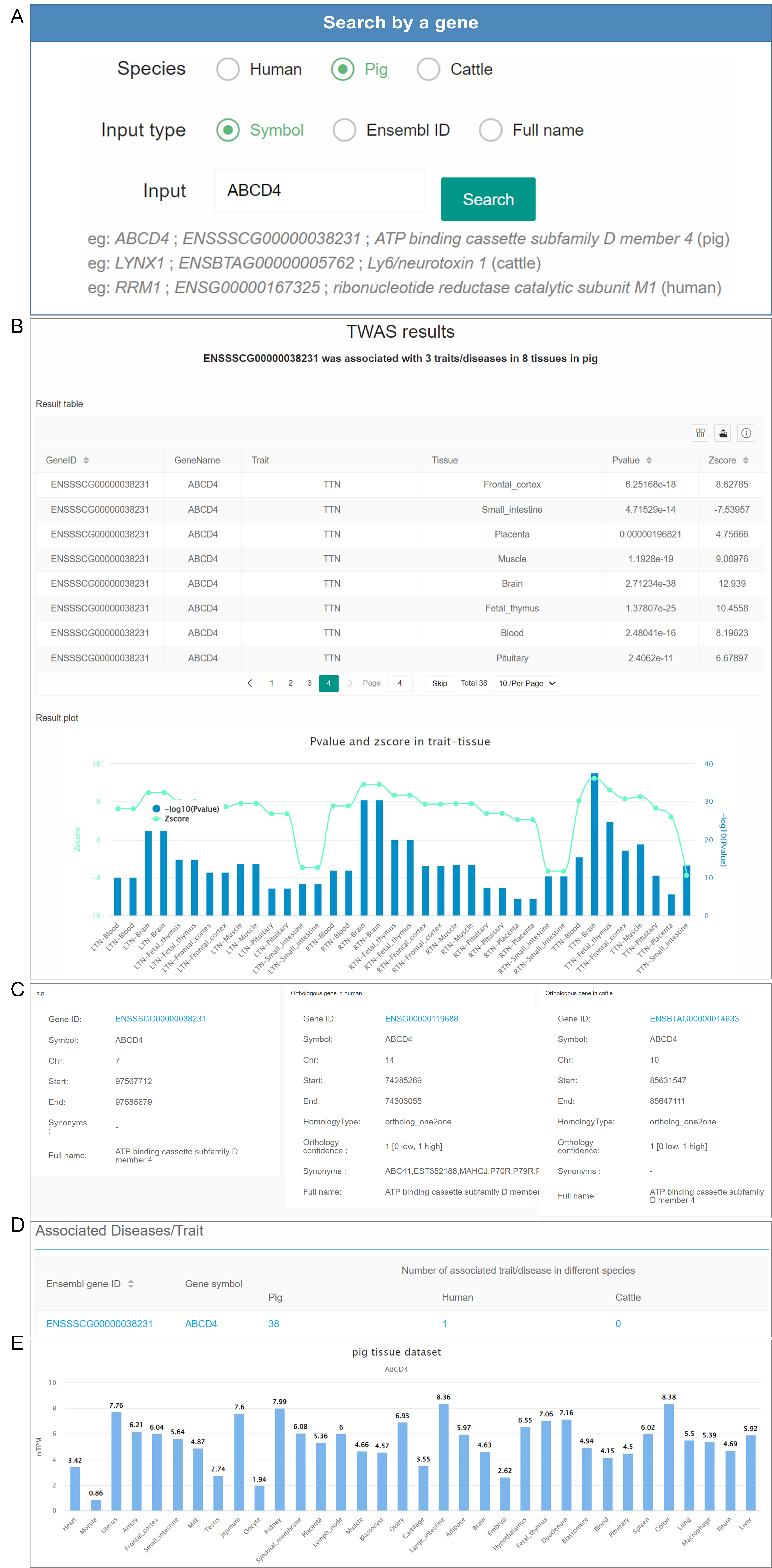

Supplement: qzaf006_Supplementary_Data [file qzaf006_supplementary_data.zip › Supplementary Figure 7.png]

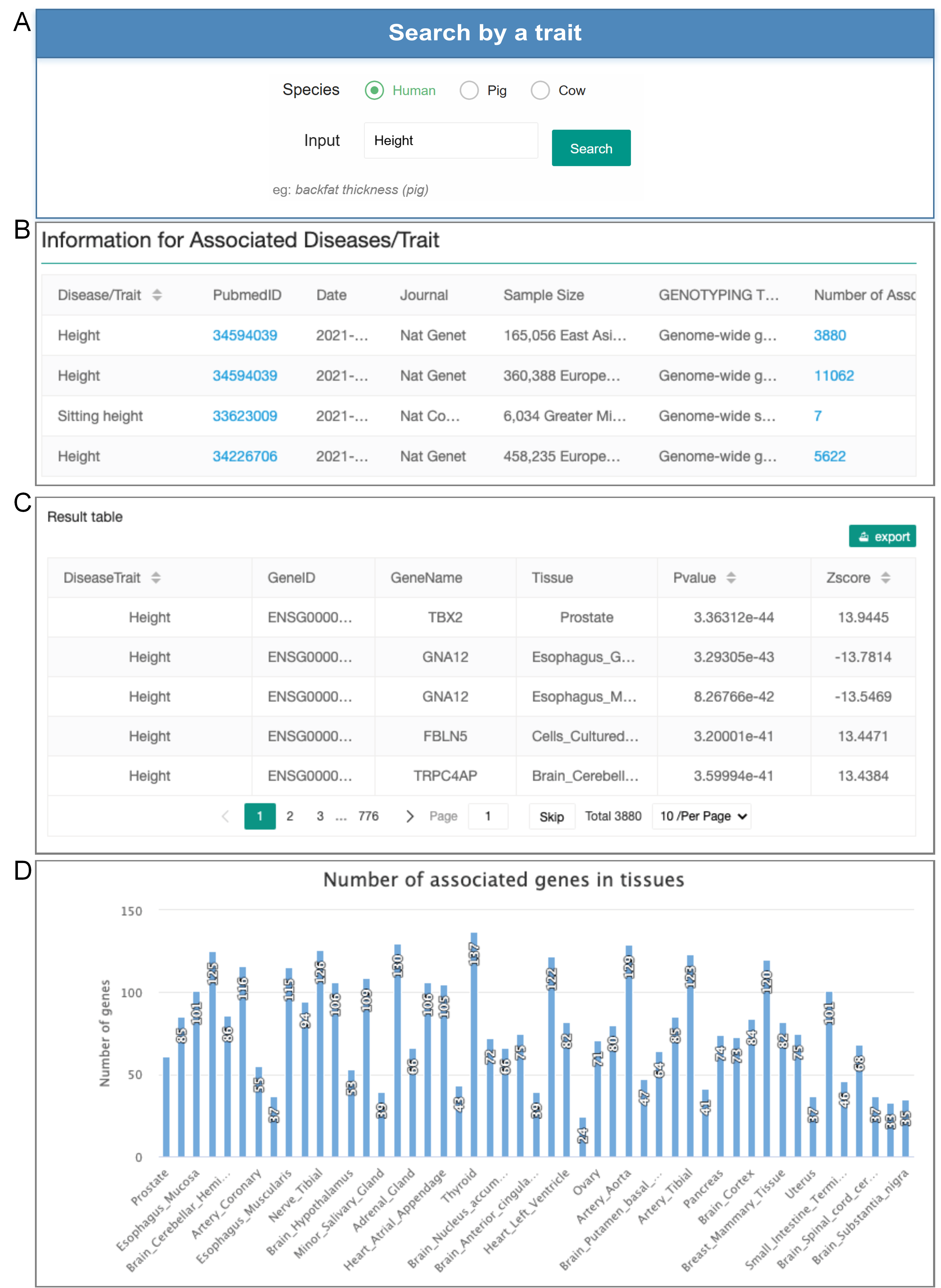

Supplement: qzaf006_Supplementary_Data [file qzaf006_supplementary_data.zip › Supplementary Figure 8.png]

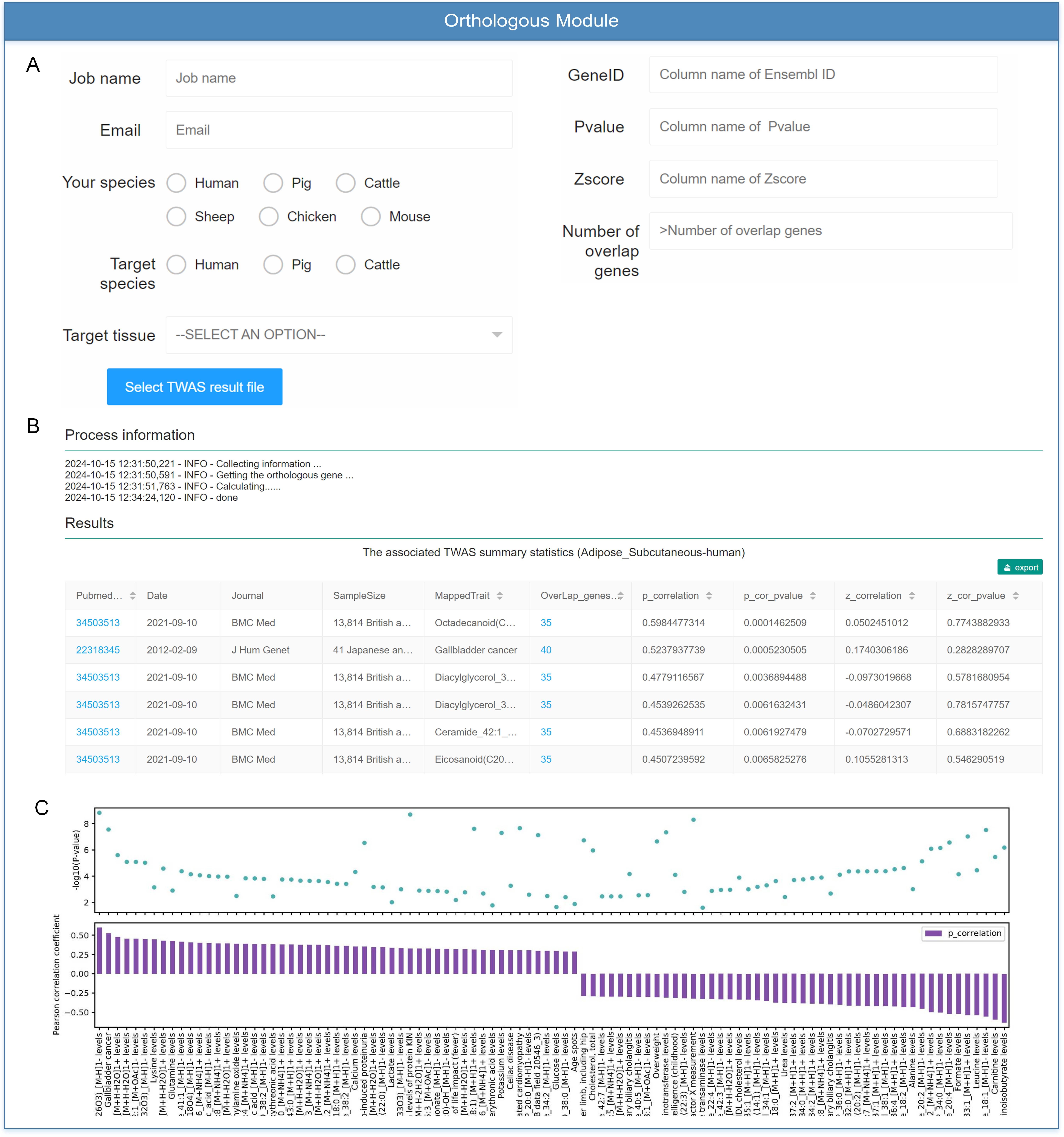

Supplement: qzaf006_Supplementary_Data [file qzaf006_supplementary_data.zip › Supplementary Figure 9.png]
